# Functional EEG connectivity in infants associates with later restricted and repetitive behaviours in autism; a replication study

**DOI:** 10.1038/s41398-019-0380-2

**Published:** 2019-02-04

**Authors:** Rianne Haartsen, Emily J. H. Jones, Elena V. Orekhova, Tony Charman, Mark H. Johnson, The BASIS team, S. Baron-Cohen, R. Bedford, A. Blasi, P. Bolton, S. Chandler, C. Cheung, K. Davies, M. Elsabbagh, J. Fernandes, I. Gammer, H. Garwood, T. Gliga, J. Guiraud, K. Hudry, M. Liew, S. Lloyd-Fox, H. Maris, L. O’Hara, G. Pasco, A. Pickles, H. Ribeiro, E. Salomone, L. Tucker, A. Volein

**Affiliations:** 1 Centre for Brain and Cognitive Development, Birkbeck College, University of London, London, WC1E 7HX United Kingdom; 2 Autism Research Laboratory, Moscow State University of Psychology and Education, Moscow, Russia; 3 Center for Neurocognitive Research (MEG Center), Moscow State University of Psychology and Education, Moscow, Russia; 4 Department of Psychology, Institute of Psychiatry, Psychology and Neuroscience, King’s College London, De Crespigny Park, London, SE5 8AF United Kingdom; 5 South London and Maudsley NHS Foundation Trust, Bethlem Royal Hospital, Monks Orchard Road, Beckenham, Kent, BR3 3BX United Kingdom; 6 Department of Psychology, University of Cambridge, Cambridge, United Kingdom

## Abstract

We conducted a replication study of our prior report that increased alpha EEG connectivity at 14-months associates with later autism spectrum disorder (ASD) diagnosis, and dimensional variation in restricted interests/repetitive behaviours. 143 infants at high and low familial risk for ASD watched dynamic videos of spinning toys and women singing nursery rhymes while high-density EEG was recorded. Alpha functional connectivity (7–8 Hz) was calculated using the debiased weighted phase lag index. The final sample with clean data included low-risk infants (*N* = 20), and high-risk infants who at 36 months showed either typical development (*N* = 47), atypical development (*N* = 21), or met criteria for ASD (*N* = 13). While we did not replicate the finding that global EEG connectivity associated with ASD diagnosis, we did replicate the association between higher functional connectivity at 14 months and greater severity of restricted and repetitive behaviours at 36 months in infants who met criteria for ASD. We further showed that this association is strongest for the circumscribed interests subdomain. We propose that structural and/or functional abnormalities in frontal-striatal circuits underlie the observed association. This is the first replicated infant neural predictor of dimensional variation in later ASD symptoms.

## Introduction

Autism Spectrum Disorder (ASD) is characterized by difficulties in social communication, atypicalities in sensory perception, and restricted and repetitive behaviours^1^. In many cases, the diagnosis can reliably be made by toddlerhood^2^. Early diagnosis and treatment might influence developmental trajectories and could significantly impact later quality of life^3,4^. To this end, it is crucial to identify early infant biomarkers that can predict diagnosis of autism and/or dimensional variation in relevant traits in later development. Moreover, research on early biomarkers can reveal underlying mechanisms and putative causal pathways to later ASD symptoms^5^. Biomarkers are objective measures that indicate typical biological processes. These markers are used for diagnosis, outcome predictions, or prediction and effect of treatment. High accuracy, reliability, and validity of these biomarkers are essential for their use in the clinical field^6,7^. Furthermore, uncertainty about psychology and neuroimaging findings is emerging as reproducibility of results in neuroscience is currently only moderate, particularly in studies were sample sizes are small^8^. To this end, replication of potential biomarkers in independent cohorts is crucial. Several electroencephalographic (EEG) measures have been suggested as potential diagnostic biomarkers, such as event-related potentials, spectral power, and functional connectivity^9^. However, these findings remain to be replicated in separate cohorts.

Functional EEG connectivity has been suggested as a fruitful source of potential biomarkers^10^. Functional connectivity indicates how different brain regions synchronize or communicate^11^. It has been suggested that ASD is characterized by atypical brain connectivity from an early age^12^. Preliminary evidence from a prospective study of infants with older siblings with ASD indicates that atypicalities in brain functional connectivity may predict later symptom emergence^10^, consistent with a putative role in causal pathways and raising the potential for predictive biomarkers.

Infants with an older sibling with ASD (‘high-risk infants’) have an approximately 20% chance of receiving an ASD diagnosis in prospective studies^2,13^, making prospective longitudinal studies of ASD emergence feasible. Using this design, we (Orekhova and colleagues) previously analysed EEG data collected from 14-month-old infants with and without older siblings with ASD (infants with a high risk (HR) and with low risk (LR), respectively^10^). Infants watched dynamic videos of spinning toys, a hand spinning toys, and women singing nursery rhymes, while high-density EEG was recorded^14^. Functional connectivity was calculated using the debiased weighted phase lag index^15^, as phase lagged connectivity would minimise the effect of volume conduction on the connectivity estimates compared to other metrics^16^.

Results showed that the infants later diagnosed with ASD at 3 years (relative to those who were not) displayed higher global EEG connectivity (also called whole brain connectivity) in the alpha frequency band (7–8 Hz) at 14 months of age. The elevated connections were particularly strong for frontal and central regions (also see Fig. 2b in ref. ^10^). The choice of the narrow alpha frequency band was dictated by presence of the peaks in the power and connectivity spectra suggesting functional significance of this frequency band, as well as its good signal-to-noise ratio. Additionally, compared to the beta and gamma bands this EEG band is less prone to contamination from myogenic activity^17,18^, which causes problems in young children and infants in particular.

We also previously examined associations between functional connectivity and dimensional measures of ASD-related traits. The etiological paths that contribute to ASD diagnosis are likely to also contribute to variation in ASD-related traits^5^. Given substantial heterogeneity in symptom profiles within groups of children with ASD, it may be more appropriate to identify biomarkers of particular dimensional traits that may relate to different underlying brain systems and indicate different profiles of subsequent clinical need. In our previous study^10^, the group of high-risk infants showed trend-level correlations between higher average global connectivity and more later restricted and repetitive behaviours (RRBs), and more severe social and the communication symptoms measured by the Autism Diagnostic Interview–Revised at age 3 years (ADI-R)^19^). There were no associations with symptoms measured on the Autism Diagnostic Observational Schedule–Generic (ADOS-G)^20^. Second, connections that showed higher connectivity in the HR-ASD group (HR infants with later diagnosis of ASD) compared to both the LR and HR-no ASD group (HR infants without later diagnosis of ASD) were selected and used to further investigate associations with dimensional measures of ASD traits. These connections were mostly located between frontal and central regions. We reported that increased connectivity in these selected fronto-central connections was significantly related to higher severity of restricted and repetitive behaviours measured by the ADI-R, but not with social communication difficulties or ADOS scores.

We speculate that associations between fronto-central connectivity and restricted and repetitive behaviours might relate to atypicalities in frontal and striatal structures^21,22^. Further, the association with RRBs could be related to the fact that we measured functional connectivity under conditions of sustained attention. One potential cognitive component of restricted and repetitive behaviours is the over-focused attention typically observed in young individuals with ASD^23^. Alpha oscillations (our frequency band of interest) are closely related to attention processes. For example, performance on visuospatial attention tasks is associated with suppression of alpha band amplitudes, and increase in the alpha band phase synchronization^24,25^, while increases in alpha amplitude during task performance may reflect active suppression of interference^26,27^. In our previous study functional connectivity in infants during sustained attention peaked in the alpha band, suggesting its functional relevance in attention processes within this experimental paradigm^16^. Thus, early elevated alpha connectivity could reflect an over-focused attentional style that is predictive of later RRBs. However, before we focus closely on mechanism we need to determine whether these findings are robust and replicable.

Our previous findings are difficult to compare to the broader literature because of differences in the methodologies used to compute connectivity and the target frequency bands across studies. For example, Domínguez and colleagues report increased functional EEG connectivity (measured with the imaginary part of coherency) in toddlers with ASD compared to those with typical development (TD) across alpha, theta, and delta bands^28^. However, Boersma and colleagues report no differences between 2 to 5-year-old toddlers with ASD and typical development in EEG connectivity (phase-lag index) over broadband (.1–30 Hz) or theta-alpha band^29^. Other studies have reported *no* intra-hemispheric functional EEG connectivity (linear coherence) differences between low-risk and high-risk infants in the gamma band^30^, and no connectivity (phase coherence) differences in 6-month-old infants^31^. Indeed, some studies report findings of *decreased* intra-hemispheric connectivity (linear coherence) in the gamma band in 12-month-old high-risk infants with a later diagnosis of ASD (relative to other high-risk or low-risk infants), and decreased connectivity (phase lag index) in toddlers with ASD (versus TD) for the beta band^29,30^. The lack of consistency across findings of functional connectivity may depend on age, task and length of the EEG recordings, frequency band of interest, the selected index of functional connectivity, and small sample sizes, among others^12,32^. Moreover, the heterogeneity in ASD and the possibility of subtypes of ASD might underlie the inconsistent findings. Focussing on replicating particular analytic approaches across studies, and looking at both categorical and dimensional levels of ASD, will be critical steps forward for this field.

In the present study, we attempted to replicate the observation of alpha hyper-connectivity in 14-month-old infants with later ASD, and the relation between alpha hyper-connectivity and emergent restricted and repetitive behaviours^10^. We choose to replicate this study in particular because of both the categorical and dimensional approaches taken that allows for investigation of the heterogeneity of ASD. Further, the measure of connectivity chosen is likely more robust to common issues like volume conduction, unequal trial numbers, and electrode bridging^15^. We studied a new cohort of 143 infants, but focussed on the same age group, paradigm, and functional connectivity measure as Orekhova and colleagues^10^. The only addition to the design was the split of the HR-no ASD group into a group with HR infants who were typically developing (HR-TD) and those who were not typically developing but did not meet the ASD criteria (HR-Atyp). We did this to investigate whether there were any differences between the HR infants who develop ASD compared to those who develop atypically but who do not have ASD (including both those with sub-threshold symptoms consistent with the broader autism phenotype (BAP) and those with language and/or developmental delay) and HR infants who are typically developing, as well as low risk controls^33–36^. Based on the findings in our previous study, we predicted that functional EEG connectivity in the alpha range would be increased particularly in the frontal and central areas for HR-ASD infants compared to the other groups. Further, we predicted that overconnectivity in the connections that distinguished HR-ASD from LR and HR-no ASD groups in our previous study^10^ would selectively associate with later severity of restricted and repetitive behaviours measured with the ADI-R.

In a second step, we combined the two cohorts in order to ask new questions about the potential nature of the observed associations between functional connectivity and RRBs. Previous studies provide evidence for three subtypes of RRBs: Repetitive Motor Behaviours, Insistence on Sameness, and Circumscribed Interests^37,38^. These are likely caused by different underlying mechanisms^39^. We tested whether associations between functional connectivity and RRBs were specific for either or several of these subtypes in order to provide more insight into potential mechanisms.

## Methods

### Participants

143 infants participated in the current study, from which 101 infants provided sufficient EEG data for further analyses. Infants were excluded due to: missing outcome data at the visit at 3 years of age (*N* = 3), no data recorded (*N* = 8: no visit *N* = 1, equipment failure *N* = 2, and 5 infants were indisposed), or artefacts in the EEG data (*N* = 31). Infants were between 13 and 18 months old. The study protocol was approved by the London Central NREC (code 06/MRE02/73; 08/H0718/76). All experiments and assessments were performed in accordance with relevant guidelines and regulations. Informed consent was obtained from the parent/ caregivers before the start of the study.

Descriptions for the previous cohort can be found in our previous report^10^ (also see S1.1).

### Materials and procedure

The current study focused on EEG collected at 13 to 18 months (mean 14 months), and data from a clinical assessment at 36 months of age.

#### EEG stimuli

**Fig. 1:**
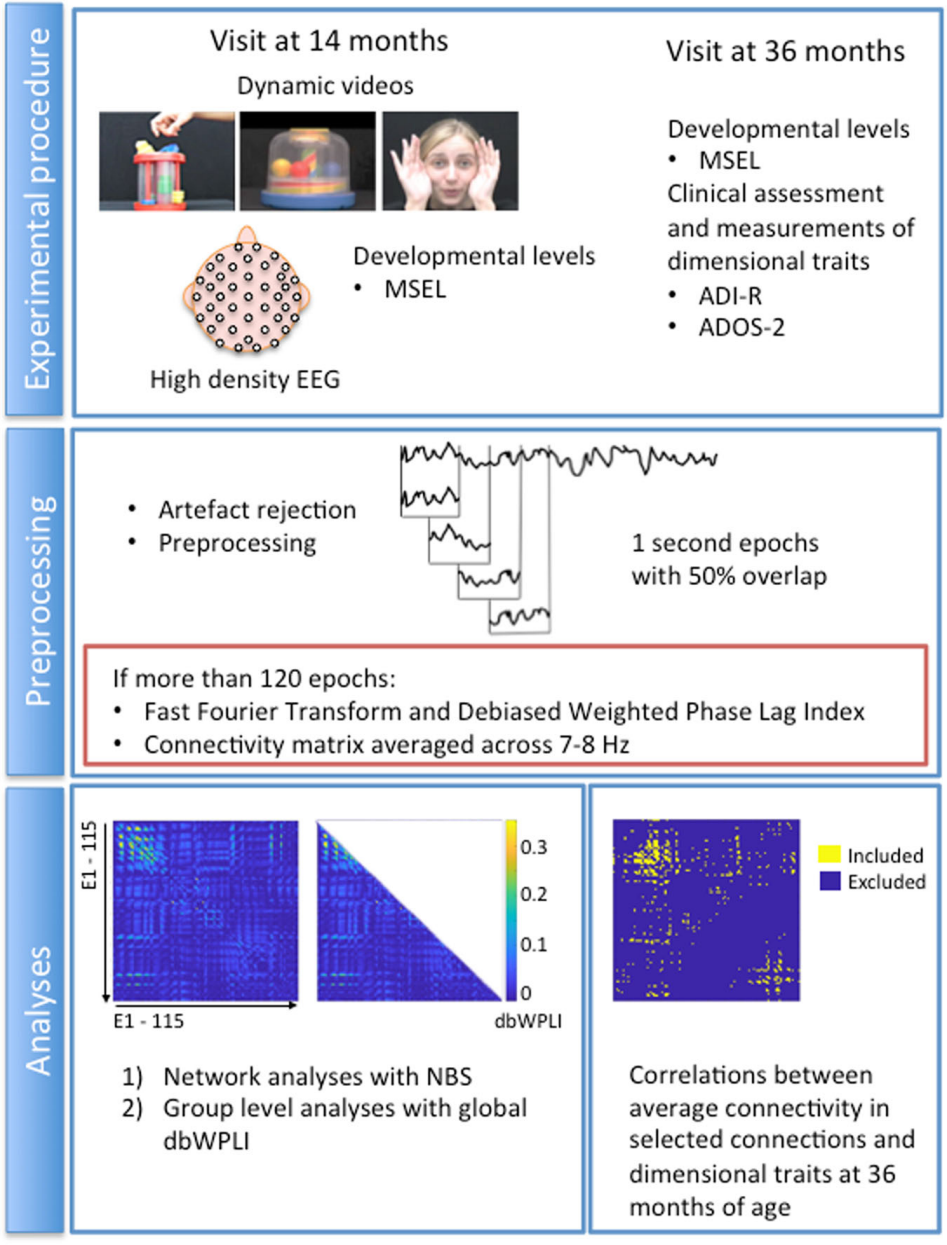
Overview of the methods used. Methods are the same as in the previous study. EEG was recorded while 14-month-old infants watched dynamic videos. At 36 months, a clinical assessment and measurements of dimensional ASD traits were performed. Developmental abilities were assessed at both visits. EEG data were cleaned, preprocessed, and cut into 1-s epochs. After Fast Fourier Transformations, the debiased Weighted Phase Lag Index (dbWPLI) was calculated. Connectivity matrices contain connectivity values for each possible connection pair. These were averaged across the frequencies for the 7–8 Hz band. We used the connectivity matrices of each infant to assess network differences between groups with the Network Based Statistics program. Global dbWPLI was calculated by averaging values below the diagonal of the connectivity matrices. These values were used to assess differences on group level. Finally, average connectivity in selected connections only (here displayed in yellow) was used to investigate the correlations between functional connectivity and dimensional traits. MSEL Mullen Scales for Early Learning, ADI-R Autism Diagnostic Interview–Revised, ADOS-2 Autism Diagnostic Observational Schedule–2, NBS Network Based Statistics, dbWPLI debiased Weighted Phase Lag Index

Infants were presented with the 3 different dynamic videos used in the original study^10,14^. These videos depict spinning toys (duration: 44 s), a hand spinning the toys around (duration: 41 s), and women singing nursery rhymes (duration: 32 s) (see Fig. 1). Infants sat on their parent’s lap in an electrically shielded room while looking at a computer screen. Infant EEG was recorded while the 3 videos were presented in a random order. These 3 videos were subsequently repeated 2 times, resulting in 3 presentations for each condition, and 9 presentations in total. The infants’ behaviour during this EEG session was recorded with a video camera.

#### Mullen scales for early learning (MSEL)

During the visit at 14 months of age, the Mullen Scales for Early Learning (MSEL^40^) was administered. The MSEL is a semi-structured behavioural assessment that investigates the infants’ abilities in fine motor skills, visual reception, receptive language, expressive language, and gross motor skills. The scores on these domains were combined and recalculated per the measure norms into composite standard scores that give a measure of the infants’ developmental level. The MSEL was also administered at the 36-month-old visit.

#### Clinical assessment

All HR toddlers received a clinical assessment at the 36-month-old visit performed by experienced clinicians. Outcome diagnosis was by clinical judgment based on the Autism Diagnostic Observation Schedule–2 (ADOS^20^), the Autism Diagnostic Interview–Revised (ADI-R^19,41^), the Vineland Adaptive Behavior Scale-II (VABS^42^), the Social Communication Questionnaire (SCQ^43^), and the scores on the Mullen Scales for Early Learning (also see S1.1.1 and S1.1.2).

#### Autism diagnostic observation schedule–2 (ADOS-2)

Toddlers were assessed with the Autism Diagnostic Observation Schedule–2 (ADOS^20^) by an experienced researcher. The ADOS-2 is a standardized observational assessment that evaluates the current level of ASD symptoms in the individual under investigation. The toddlers’ responses and behaviour during this session are rated on 2 scales: (1) Social Affect, and (2) Restricted and Repetitive Behaviour. These scales were used as measures of dimensional traits of ASD and included in analyses for associations between functional connectivity and dimensional ASD traits.

#### Autism diagnostic interview–revised (ADI-R)

The Autism Diagnostic Interview–Revised (ADI-R^19,41^) was conducted as an interview with the parents or caregivers of the toddler. The semi-structured interview includes 93 items that investigate ASD symptoms across several domains. Scores are given for the severity of current symptoms happening over the last 3 months (current scores) and severity of symptoms happening over the entire course of the toddler’s life (ever scores). These scores are used to calculate algorithm for ‘the Communication Algorithm Total’, ‘the Social Algorithm Total’, and ‘Behaviours/Repetitive Interests Algorithm Total’. The scores were used as measures of dimensional traits for analyses of associations between functional connectivity and dimensional traits. Scores for subtypes of restricted and repetitive behaviours were computed by summing up raw ever scores for specific items on the ADI-R (see S1.1.4).

### EEG recording and preprocessing

EEG recording and preprocessing steps were same as reported in ref. ^10^ (also see S1.2 for a more detailed description). Infants’ EEG was recorded with a 128 channel EGI electrode system and Netstation EGI software at a sampling rate of 500 Hz (Electro Geodesics, Inc., Eugene, USA). Data were preprocessed using FieldTrip^44^ and MATLAB_R2015a (MathWorks, Natick, USA). Video recordings from the EEG session were coded for attention and interference (e.g. parent or experimenter is talking, parent or experimenter is pointing to the screen, parent is stroking the infant) by RH and a research volunteer. Inter-rater reliability was high for looking (Spearman’s rho = .90) and moderate for interference (Spearman’s rho = .73) for the double-coded videos of 12 infants that were randomly chosen from the complete sample.

EEG segments were excluded from further analyses when the infant was not paying attention, interference occurred, or when the segment contained artefacts. After manual and automatic artefact rejection, the remaining data segments were cut into 1-s epochs with 50% overlap. Infants with more than 120 epochs across all three conditions were included in further analyses. Fast Fourier Transform (FFT) was performed after a Hanning window was applied to each of the clean epochs. The Fourier transformed data were used for functional connectivity analyses and spectral power analyses.

Functional connectivity was measured with the debiased weighted phase lag index (dbWPLI) that was calculated for each possible pair of connections from the FFT values^15,16^. Values close to 0 reflect low connectivity whereas values closer to 1 reflect high connectivity. The functional connectivity matrices were averaged across frequencies for 7–8 Hz. Global dbWPLI values were calculated by averaging dbWPLI values for all possible pairs of connections. We also calculated functional connectivity in the selected connections by averaging dbWPLI values for those connections that separated HR-ASD infants from the control comparison groups in the previous study only (see Fig. 1).

### Statistical analyses

Measures of behaviour and functional connectivity for different groups were tested for normality and homogeneity of variance with a Shapiro-Wilk test and a Levene’s test, respectively. If the assumptions for normality and homogeneity were both met, parametric independent samples *t*-tests for means or Pearson’s correlations were applied. In the other cases, a non-parametric Mann–Whitney *U*-test or Spearman’s correlation was applied for comparisons between the HR-ASD and other groups. These tests were performed with the Statistical Package for Social Sciences (IBM SPSS Statistics, version 22). This procedure of analyses was also applied in the previous study^10^ (also see S1.2.5).

Functional connectivity analyses consisted of 3 parts: First, testing for group differences in networks: Network Based Statistics (NBS^45^) was applied to the connectivity matrices to test for significant network differences between the HR-ASD and comparison groups. The Mann–Whitney *U*-test NBS version is a non-parametric permutation-testing program that circumvents the multiple comparisons problem. Mann–Whitney *U*-tests were one-tailed, alpha significance level was 0.05, Z-score threshold was set to 1.96, and the number of permutations was 5000. Statistical values and *p*-values are only reported in the output if *p*-values are below the alpha significance level, otherwise the NBS program output contains no results. Second, testing for group differences in global functional connectivity: normality and homogeneity of global functional connectivity data were tested with a Shapiro-Wilk test and a Levene’s test, respectively. If the assumptions were met, a parametric independent samples t-test was used, whereas a non-parametric Mann–Whitney *U*-test was used in other cases (two-sided tests). Third, testing for group differences in global functional connectivity while correcting for confounding factors: in the event of significant differences between groups for age, gender, behaviour during the EEG recording, and spectral power, additional analyses were used to account for these factors or covariates with an Analysis of Variance (ANOVA) or General Linear Model (GLM), respectively. Only the first and second parts of the analyses are reported here. The third part is reported in the S2.

Finally, correlations between functional connectivity and dimensional ASD traits were assessed using Spearman’s rank correlations^46^. Correlations for both global functional connectivity across all channels and functional connectivity in the set of selected connections from the previous study with dimensional traits were calculated. Dimensional traits were measures with 4 scales: (a) the ADI-R Social and Communication Algorithm Total 36 m, (b) the ADI-R Behaviour/ Repetitive Interests Algorithm Total 36 m of the ADI-R (ADI-R RRB), (c) the ADOS-2 Social Affect Total 36 m, and (d) the ADOS-2 Restricted and Repetitive Behaviors Total 36 m (ADOS RRB). We did not correct for multiple comparisons for the analyses where we had a priori hypotheses (overall HR group, and HR-ASD group), but did use a correction for those where we did not have a specific a prior hypothesis (HR-TD and HR-Atyp groups). In the latter case, we used the False Discovery Rate method (FDR^47^) to correct for multiple comparisons within each group. Furthermore, explanatory analyses were performed after combining data from the previous and current cohort with subtypes of restricted and repetitive behaviours measured by the ADI-R: (1) Repetitive Motor Behaviours, (2) Insistence on Sameness, and (3) Circumscribed Interests. To increase statistical power, we collapsed data from the current and our previous study for these exploratory analyses only. We also corrected for multiple comparisons here using the FDR method.

### Data and code availability

The datasets analysed during the current study and Matlab codes used to analyse the data are available from the corresponding author upon reasonable request.

## Results

### Demographics

The methods and analyses performed in the current study were identical to the ones used by Orekhova and colleagues, where measures were compared between the HR-ASD and other groups (also see Fig. 1, and S1). The final sample consisted of 20 LR infants (11 males), 47 HR-TD infants (22 males), 21 HR-Atyp infants (14 males), and 13 HR-ASD infants (11 males; see Table 1). Groups were matched in age but as expected the distribution of gender in the HR-ASD group (more males than females) was different from the distribution in the LR and HR-TD group, but similar to the HR-Atyp group. Although age was not related to functional connectivity in the complete sample, the correlations between age and functional connectivity were significant (*p*’s ≤ 0.023) in the HR-TD and HR-Atyp group (see S 2.3). As expected, composite standard scores for the Mullen Scales for Early Learning (MSEL^40^) at 14 and 36 months were higher for the LR and HR-TD group than the HR-ASD group, whereas the HR-Atyp and HR-ASD group showed no significant difference.

**Table 1:** Demographics of the final sample in the current cohort

	LR	HR-TD	HR-Atyp	HR-ASD
Number of participants (male)	20 (11)	47 (22)	21 (14)	13 (11)
	*χ*^2^(1) = 3.11,	*χ*^2^(1) = 5.88,	*χ*^2^(1) = 1.33,	
	*p* = 0.078^a^	*p* = 0.015	*p* = 0.249	
Age at EEG assessment, in days	473 (49)^b^	470 (41)	465 (46)	446 (57)
	*U* = 91.5,	*U* = 234.5,	*U* = 113,	
	*p* = 0.158^c^	*p* = 0.203^d^	*p* = 0.420^c^	
Age at diagnostic assessment, in months	38.0 (1.0)^b^	39.0(1.3)	38.0 (2.0)	38.5 (1.0)
	*U* = 106,	*U* = 230.5,	*U* = 118.5,	
	*p* = 0.950^c^	*p* = 0.369^d^	*p* = 0.782^c^	
MSEL^e^ Composite standard score at visit at 14 months	102 (14)^f^	98 (12)	93 (16)	87 (13)
	81–133^g^	71–121	67–123	65–113
	*t*(31) *=* 3.10,	*t*(58) *=* 2.96,	*t*(32) = 1.12,	
	*p* = 0.004	*p* = 0.004	*p* = 0.270	
MSEL^e^ Composite standard score at visit at 36 months^h^	123 (15)^b^	115 (20)	83 (26)	78 (40)
	69–137^g^	79–142	54–145	49–142
	*U* = 37,	*U* = 103.5,	*U* = 107,	
	*p* = 0.002^c^	*p* = 0.001 ^d^	*p* = 0.494^c^	
ADI-R Social total^h,i^	1 (2)^b^	1 (2)	2 (3)	13 (5)
	0–6 ^g^	0–11	0–10	2–25
	*U* = 6,	*U* = 16.5,	*U* = 13,	
	*p* < 0.001^c^	*p* < 0.001^d^	*p* < 0.001^c^	
ADI-R, Communication total^h,j^	0 (1)^b^	1 (3)	3 (6)	12 (5)
	0–4 ^g^	0–11	0–14	4–19
	*U* = 0.5,	*U* = 17,	*U* = 25,	
	*p* < 0.001 ^c^	*p* < 0.001 ^d^	*p* < 0.001 ^c^	
ADI-R	0 (0)^b^	0 (1)	1 (2)	6 (4)
RRB total^h,k^	0–1 ^g^	0–3	0–9	0–10
	*U* = 10,	*U* = 35.5,	*U* = 32,	
	*p* < 0.001^c^	*p* < 0.001^d^	*p* < 0.001^c^	
ADOS-2,	2.5 (5)^b^	1 (1)	6 (7)	5 (6)
Social affect total^h,l^	0–9^g^	0–5	0–13	1–12
	*U* = 68,	*U* = 111.5,	*U* = 109,	
	*p* = 0.095^c^	*p* = 0.001^d^	*p* = 0.542^c^	
ADOS-2	1 (1)^b^	1(1)	2 (2)	1 (3)
RRB total^h,m^	0–3^g^	0–3	0–6	1–6
	*U* = 57,	*U* = 141,	*U* = 124.5,	
	*p* = 0.031^c^	*p* = 0.006^d^	*p* = 0.956^c^	

^a^Pearson Chi-Square with asymptotic significance values (2-sided)

^b^Medians and interquartile range in parentheses, with results for the Mann–Whitney *U*-test when compared with the HR-ASD group

^c^Exact 2-tailed

^d^Asymptotic 2-tailed

^e^Mullen Scales for Early Learning (MSEL)

^f^Means and standard deviations in parentheses, with results for the *t*-test for independent samples when compared with the HR-ASD group

^g^Range with minimum and maximum score

^h^Data for the 36-month-old visit was only available for 18 LR infants, 46 HR-TD infants, 21 HR-Atyp infants, and 12 HR-ASD infants

^i^Autism Diagnostic Interview–Revised, Social Algorithm Total at 36 months

^j^Autism Diagnostic Interview–Revised, Communication Algorithm Total at 36 months

^k^Autism Diagnostic Interview–Revised, Behaviours/ Repetitive Interests Algorithm Total 36 months

^l^Autism Diagnostic Observation Schedule–2, Social Affect Total 36 months

^m^Autism Diagnostic Observation Schedule–2, Restricted and Repetitive Behaviours Total 36 months

### Functional EEG connectivity and categorical outcome

**Fig. 2:**
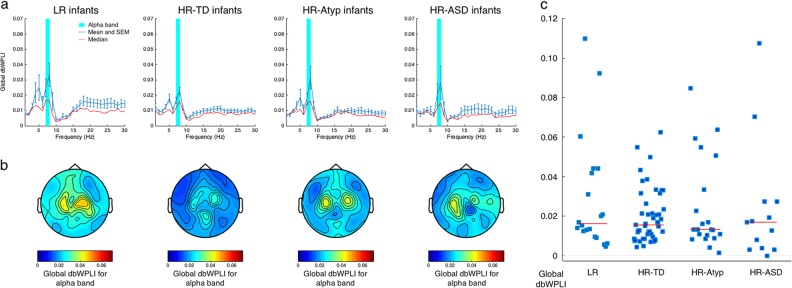
Functional EEG connectivity and categorical outcome. **a** Mean (standard error of the mean, in blue) and median (red) global dbWPLI (averaged across all electrodes) for each group for 0 to 30 Hz. The alpha band (7–8 Hz) is highlighted in cyan. **b** Topoplots for global dbWPLI across the alpha band (average connectivity for one electrode with all other 115 electrodes) for each group. **c** Global dbWPLI for the alpha band (7–8 Hz) for each group. Each square represents an individual participant. Red horizontal lines display group medians. *N*_LR_ = 20, *N*_HR-TD_ = 47, *N*_HR-Atyp_ = 21, and *N*_HR-ASD_ = 13

Based on previous findings, we expected to find higher connectivity in the HR-ASD group relative to the LR, HR-TD, and HR-Atyp groups. Functional connectivity across frequencies, and topoplots and individual functional connectivity values for the alpha frequencies are depicted in Fig. 2. Following Orekhova and colleagues^10^, we compared the networks between the HR-ASD and comparison groups using the Network Based Statistics (NBS) method with the non-parametric Mann–Whitney *U*-test^45^. The NBS method identifies any networks that yield significant differences between groups or conditions. No significant increases in the networks in the alpha range were found for HR-ASD versus LR infants, HR-ASD versus HR-TD infants, HR-ASD versus HR-Atyp infants, or HR-ASD versus HR-no ASD infants (HR-TD and HR-Atyp infants combined into one group).

Second, we computed global functional connectivity values as the mean connectivity value across all possible electrode pairs. A Mann–Whitney *U*-test was used to test whether there were any differences between the HR-ASD group and the comparison groups, since the assumptions for normality (*p’*s ≤ 0.001) and homogeneity of variance (*p* = 0.007) were not met. The comparisons between the HR-ASD groups and the comparison groups yielded no significant differences between the groups: LR versus HR-ASD: *U* = 104, *z* = −0.958, exact 2-tailed *p* = 0.353, *r* *=* −0.17; HR-TD versus HR-ASD: *U* *=* 275, *z* = −0.547, asymptotic 2-tailed *p* = 0.584, *r* *=* −0.07; HR-Atyp versus HR-ASD: *U* = 125, *z* = −0.408, exact 2-tailed *p* = 0.701, *r* *=* −0.07; *Mdn*_LR_ = 0.0162, *IQR*_LR_ = 0.0335; *Mdn*_HR-TD_ = 0.0156, *IQR*_HR-TD_ = 0.0180; *Mdn*_HR-Atyp_ = 0.0133, *IQR*_HR-Atyp_ = 0.0327; and *Mdn*_HR-ASD_ = 0.0170, *IQR*_HR-ASD_ = 0.0241. Similar results were obtained when the HR-ASD group was compared with the HR-no ASD group (HR-TD and HR-Atyp group combined, see S2.4) and when only selected connections from the previous study were used (see S2.5).

Third, we conducted additional analyses taking into account age, MSEL scores, proportion of epochs from the social condition, and gender. The results of these analyses are reported in (see S2.3). Global functional connectivity was similar across groups after controlling for each of these factors.

Of note, infants in the HR-ASD group spent an equal percentage of time looking at the screen, and experienced an equal percentage of interference from parent or experimenter during the EEG session when compared to the other groups (see Table S7). Furthermore, the quantity of epochs included in analyses for the infants did not differ between groups. Grand average alpha power (7–8 Hz) in HR-ASD infants was similar to the power levels in the comparison groups (see S2.1 and 2.2 for more details). Thus, group comparisons for connectivity cannot be confounded by differences in behaviour during EEG, the number of epochs included, or spectral power.

In summary, we did not replicate the previous observation of elevated connectivity in high-risk infants with later ASD.

### Functional connectivity and dimensional traits

We then examined the relation between functional connectivity and dimensional ASD-related traits. Both ADI-R and ADOS-2 data were missing for 1 HR-TD and 1 HR-ASD infant at the 36-month visit. The final sample for the brain behaviour correlations consisted of 46 HR-TD infants, 21 HR-Atyp infants, and 12 HR-ASD infants.

**Fig. 3:**
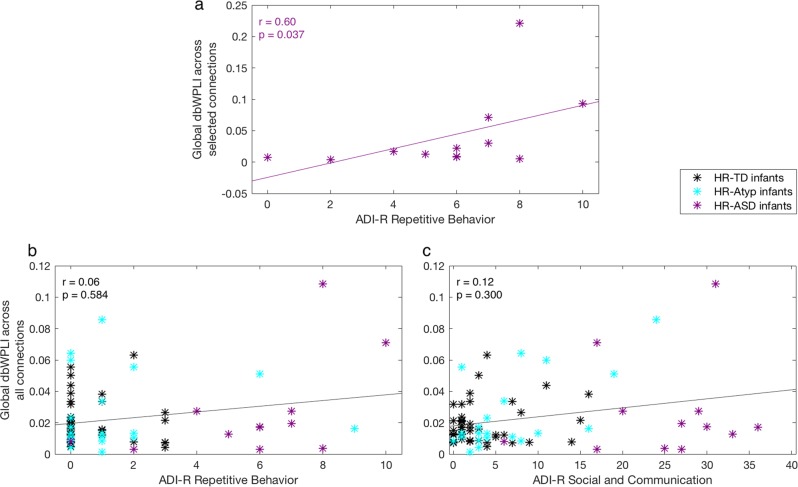
Correlations between global connectivity and dimensional traits. **a** Global connectivity among selected connections and scores on the ADI-R RRB total at 36 months of age for HR-ASD infants only. **b** Global connectivity across all connections and scores on the ADI-R RRB total at 36 months of age for the complete HR sample. **c** Global connectivity across all connections and scores on the ADI-R Social and Communication Scale Total at 36 months of age for the complete HR sample. Each asterisk represents one infant: black for HR-TD infants, cyan for HR-Atyp infants, and purple for HR-ASD infants. R and p values in the left upper corners reflect values for the lines in the scatterplots

First, Orekhova and colleagues reported an association between over-connectivity and higher scores on the ADI-R RRB scale within the HR-ASD group that was particularly strong for a selected set of fronto-central connections based on findings of group differences in connectivity. We thus attempted to replicate this pattern using both global connectivity and connectivity within the selected fronto-central connections found in the previous study^10^ using Spearman’s correlations within the HR-ASD sample. In the current HR-ASD sample, a trend correlation was found between global connectivity and the ADI-R RRB scores (Spearman’s *rho* = 0.52, *p* = 0.086, see Table 2). The correlation between global connectivity and the other symptom severity scales did not approach significance (*p*’s ≥ 0.526); ADOS-2 RRB, ADOS-2 Social Affect total, or ADI-R Social and Communication scale total. For the fronto-central selected connections, the correlation between connectivity and ADI-R RRB total was significant within the HR-ASD group (Spearman’s *rho* = 0.60, *p* = 0.037, see Fig. 3a). This correlation remained significant when replacing the highest functional connectivity value for the next highest value (winsorizing;^46^ Spearman’s *rho* = 0.61, *p* = 0.035), and showed a trend when this participant was removed from the sample (Spearman’s *rho* = 0.54, *p* = 0.088). As in the previous paper, the correlations between fronto-central connectivity and the other symptom severity scales did not reach significance (*p*’s ≥ 0.211). These findings replicate the previous results in a separate group of infants.

**Table 2:** Associations between functional connectivity and dimensional traits in the current cohort

	Dimensional trait scale	Global connectivity across all channels	Global connectivity across selected channels
*HR-ASD infants N* *=* *12*	ADI-R Social and	*r* *=* 0.20,	*r* = 0.39,
	communication^a^	*p* = 0.526	*p* = 0.211
	ADI-R	*r* = 0.52,	***r*** **=** **0.60**^b,c^
	RRB^d^	*p* = 0.086	***p*** ***=*** **0.037**
	ADOS-2	*r* *=* –0.09,	*r* = –0.01,
	Social affect^e^	*p* = 0.776	*p* = 0.983
	ADOS-2	*r* = –0.06,	*r* = 0.08,
	RRB^f^	*p* = 0.847	*p* = 0.797
*All HR infants N* *=* *79*	ADI-R Social and	*r* = 0.12^g^,	*r* = 0.03,
	communication	*p* = 0.300	*p* = 0.788
	ADI-R	*r* = 0.06^g^,	*r* = –0.02,
	RRB	*p* = 0.584	*p* = 0.890
	ADOS-2	*r* = –0.07,	*r* = –0.05,
	Social affect	*p* = 0.523	*p* = 0.664
	ADOS-2	*r* = 0.13,	*r* = 0.13,
	RRB	*p* = 0.254	*p* = 0.259

Spearman’s rho values are represented by r. *P*-values are 2-tailed. The correlations are given for functional connectivity calculated across all channels and across the selected channels. Correlations reaching significance in the current, as well as previous cohort printed in bold

^a^Autism Diagnostic Interview–Revised, sum of the Social Algorithm Total and Communication Algorithm Total at 36 months

^b^Correlations expected to reach significance based on the previous study

^c^Correlation reaching significance at 0.05 significance level (uncorrected for multiple comparisons)

^d^Autism Diagnostic Interview–Revised, Behaviours/ Repetitive Interests Algorithm Total 36 months

^e^Autism Diagnostic Observation Schedule–2, Social Affect Total 36 months

^f^Autism Diagnostic Observation Schedule–2, Restricted and Repetitive Behaviours Total 36 months

^g^Correlations expected to show a trend based on the previous study

Second, the previous study found trend-level correlations in the overall HR sample for global connectivity with ADI-R RRB, and ADI-R Social and Communication composite scores, whereas correlations with ADOS-2 RRB and ADOS-2 Social Affect did not reach significance. In the current overall HR sample (*N*_HR_ = 79), there were no significant correlations between global connectivity and the ADOS-2 RRB, ADOS-2 Social Affect, the ADI-R RRB (Fig. 3b and Table 2), or ADI-R Social and Communication scale (*p*’s ≥ 0.254) (Fig. 3c and Table 2). Nor were any significant correlations found between global connectivity among selected connections and the measures of ASD symptom severity (*p*’s ≥ 0.259) (Table 2).

The previous study found no correlations reaching significance in the HR-no ASD group. In the current cohort, no associations reaching significance were observed for the HR-TD and HR-Atyp group separately (see S2.6). The use of ADOS-G Algorithm scores as in the previous study^10^ instead of ADOS-2 scores did not change the results (see S2.7). Lastly, no correlations with subscales of the MSEL were observed (see S2.8).

In summary, we replicate the finding of an association between elevated connectivity over fronto-central connections based on the previous study and later restrictive and repetitive behaviours in infants with later ASD in the current independent cohort. We did not replicate the findings of associations between global connectivity and ADI-R RRBs, and social and communication scores in the overall HR group. Neither study however found associations with the ADOS scales in the overall HR group.

### Functional connectivity and subtypes of restricted and repetitive behaviours

Since the observation of a relation between higher functional connectivity over selected connections and restricted and repetitive behaviours was replicated in the present sample, we capitalised on the ability to combine our samples (characteristics of the previous cohort can be found in ref. ^10^) to ask how the association might differ between subtypes of these behaviours: Repetitive Motor Behaviours, Insistence on Sameness, and Circumscribed Interests^38^ (see S1.1.4). We believe that focussing on the whole HR sample here as opposed to the HR-ASD infants only would increase power to detect associations with subtypes of RRBs. We examined ‘ever’ scores that rate the highest symptom severity during the child’s life across these three subtypes, as these scores were also used to calculate scores on the ADI-R Behaviours/Repetitive Interests Total scale. Results for the combined overall HR sample are reported in Table 3 (also see S1.1.4 and S2.9 for analyses in separate cohorts, and HR-ASD and HR-no ASD groups separately). Results show that associations between connectivity both across all connections and across selected connections from the previous study, and circumscribed interests reached significance. In summary, we observed associations between alpha EEG connectivity at 14 months, and circumscribed interests at age 3 years in the combined cohorts.

**Table 3:** Relationship between functional connectivity and subtypes of restricted and repetitive behaviours from the ADI-R at 36 months for the combined HR sample of the previous and current cohort

Subtypes of RRB on the ADI-R		Global connectivity across all channels	Global connectivity across selected channels
Repetitive motor behaviours	*N*_HR_ = 103	*r* = 0.15,	*r* = 0.15
		*p* = 0.126	*p* *=* 0.126
Insistence on sameness	*N*_HR_ = 102	*r* = 0.14,	*r* = 0.07,
		*p* = 0.153	*p* = 0.500
Circumscribed interests	*N*_HR_ = 90	***r*** **=** **0.26**,	***r*** **=** **0.30**,
		***p*** **=** **0.015**	***p*** **=** **0.004**

Spearman’s rho values are represented by *r*. *P*-values are 2-tailed. Correlations reaching significance after FDR correction across the 6 comparisons are printed in bold.

*RRB* restricted and repetitive behaviours, *ADI-R* autism diagnostic interview–revised

## Discussion

The current study aimed to replicate previously reported associations between higher EEG functional connectivity in 14-month-old infants, and later diagnosis of ASD and dimensional variation in restrictive and repetitive behaviours^10^. Our results partially replicate previous reports. Specifically, we did not replicate the observation of increased functional connectivity in the alpha range in the HR-ASD group for either global connectivity, or selected fronto-central connections. Nor did we replicate the trend level correlations between global functional connectivity and ASD symptoms measured by the ADI-R in the whole HR sample. However, we did replicate the significant correlation between higher functional connectivity over fronto-central regions, and the later severity of restricted and repetitive behaviours (RRBs) measured by the ADI-R within the group of infants with later ASD. Further, by combining the two samples we showed that functional connectivity across selected channels was specifically associated with circumscribed interests and not repetitive motor behaviour or insistence on sameness. Our findings are important both in terms of the failure to replicate effects in terms of categorical outcomes, and the replication of observed effects at a dimensional level. Conducting and full reporting of replication studies in independent cohorts sets a new standard for our field.

### Brain connectivity and categorical outcome

We did not replicate previous observations of hyper-connectivity in the alpha range for infants later diagnosed with ASD at the group level^10^. Reports of altered EEG connectivity are highly inconsistent within the ASD literature, and several other studies report null effects in toddlers^29,48^ and from infancy to adolescence^49^. Whilst heterogeneity in approach, population and analytic method could explain inconsistencies in previous work, our present failure to replicate findings at a group level using identical techniques including recruitment, and experimental and analyses methods is evidence that functional connectivity in the alpha band assessed using our present protocol is either not a strong candidate biomarker for categorical ASD, or is a only a feature of a sub-set of infants that go on to later diagnosis.

Our failure to observe altered connectivity using our present protocol does not rule out the possibility of atypicalities that could be detected through other methods. For instance, fNIRS methods provided evidence of atypical connectivity in 12-month-old infants at risk for ASD compared to infants with low risk^50^, and fMRI methods measuring functional connectivity in 6-month-old infants can predict later ASD diagnosis^51^. Nonetheless, the high temporal resolution of EEG connectivity provides an important measure of connectivity. Phase lagged measures such as the dbWPLI used here are also more likely to pick up on ‘true’ connectivity differences compared to other EEG measures of connectivity that are more influenced by volume conduction and the magnitude of the signal. However, the weighting we used removes the effect of small phase lags, thereby minimizing both volume conduction effects, and potential short-range connectivity with small phase lags. It is possible that differences between outcome groups exist for connectivity with small phase lags that were underestimated by the dbWPLI.

Other possible explanations to consider are intra- and interindividual variability. Intra-individual variability in connectivity within a session may constrain our ability to capture a stable marker of trait connectivity. Frequent short fluctuations in connectivity might be related to connectivity calculated over longer durations in EEG^52,53^, As for inter-individual variation, it is widely accepted that there is substantial heterogeneity in the genetic and environmental risk factors for ASD^54^. Analyses of large cohorts have indicated inter-individual variability in early cognitive and symptom trajectories^55–57^. Further, MEG and fMRI studies have shown that inter-individual variability in brain development and brain activation patterns in ASD is high^58–60^. The same likely applies to the current cohorts. Indeed, careful inspection of the individual data from Orekhova’s study and the current study reveals that inter-individual variation is also evident in HR-ASD infants. The sample in Orekhova’s study contained 6 infants with very high connectivity levels, whereas 4 other infants had lower connectivity levels. In contrast, the current sample contained 2 out of 13 infants displaying high levels of connectivity. Thus, the stratification of HR-ASD infants into subtypes of ASD based on functional connectivity in the alpha range at 14 months of age could be used to determine whether these represent distinct ‘subtypes’ of ASD. In relatively modest sample sizes there will be stochastic variation in the proportion of the sample showing elevated connectivity, creating difficulties in replicability at the group level.

### Functional connectivity and the severity of restricted and repetitive behaviours

The heterogeneity poses a genuine challenge for research in ASD and some researchers even question the validity of ASD as a construct itself^61^. One potential solution to manage this is to take a dimensional approach in addition to a categorical classification and look at specific core ASD symptom domains and brain-behaviour associations as a diagnosis of ASD can be reached by multiple different combinations of specific symptoms^62,63^. To this end, we focussed on investigating associations between functional connectivity and ASD core symptoms measured by the ADI-R and ADOS-2. Replicating Orekhova and colleagues^10^, we found a significant relationship between functional connectivity averaged across selected connections from the previous study and the severity of RRBs within the HR-ASD group. Overall, our findings suggest that the heterogeneity in brain mechanisms is associated with specific heterogeneity in the behavioural phenotype. The absence of significant findings based on categories and the presence of significant brain-behaviour associations support the notion that a dimensional perspective should be taken when considering ASD, rather than solely a categorical approach^5^. To our knowledge, this result is the first replication of an infant neural predictor of dimensional variation in later ASD symptoms.

What mechanism underlies our replicated association between functional connectivity in fronto-central regions and later RRBs? The high alpha connectivity in fronto-central regions in HR-ASD infants in our study could contribute to the observed behavioural abnormalities and/or reflect common pathological factors affecting both EEG and behaviour, such as e.g. functional/neurochemical abnormalities^64–68^, and structural changes observed in frontal and related subcortical areas in ASD (^69,70^, also see^71–74^). Of note, it has been found that severity of RRBs in older children and adults with ASD correlated with frontal and/or striatal neurochemical abnormalities^64,66^, and structural changes in frontal cortex and related subcortical areas (such as the cerebellum, and caudate nuclei)^69^.

Studies of structural connectivity in infants at high familial risk for ASD show a somewhat converging pattern of findings in relation to RRBs. Specifically, cortical area and cortical thickness of the corpus callosum during the first year of life were positively associated with later RRBs in HR-ASD infants^75^. Further, higher structural connectivity between the genu of corpus callosum and the cerebellum at 6 months of age was related to more severe higher order RRBs (such as rituals, compulsions, insistence on sameness, and circumscribed interests) at later age^76^. The genus of the corpus callosum plays an important role in the frontal-striatal circuits. It has been suggested that fronto-striatal circuits might be implicated in the underlying mechanisms of RRBs^39,77,78^, occurring in ASD but also in for example obsessive compulsive disorder.

Further, analyses conducted on the combined high-risk sample suggest these findings arise from associations between functional connectivity in the alpha frequency EEG and circumscribed interests that can be detected at the trait level in the high-risk group as a whole. This observation seems consistent with the idea that elevated alpha connectivity reflects an over-focused attentional style, which is more closely related to circumscribed interests than the other subtypes of RRBs. Possibly, we did not observe an association between alpha connectivity and RRBs in the high-risk group because the circumscribed interests are the driving factor, and might not be strong enough to show an association with alpha connectivity when combined with the other RRB subtypes. Langen and colleagues^39^ have proposed that circumscribed interests are mediated by a limbic loop consisting of the anterior cingulate cortex (ACC), orbitofrontal cortex (OFC), ventral striatum, ventral palladium, and medial dorsal thalamic nucleus. Our observation of functional hyperconnectivity over frontal and central scalp regions would at least be consistent with functional changes in the cortical part of this loop, though source analysis combining MRI and EEG techniques would be required to confirm this.

Finally, the association between functional connectivity for selected connections with RRBs should be taken with consideration of the measurements of RRBs. The association between functional connectivity and RRBs reached significance when measured with the ADI-R, but not with the ADOS-2, and only in the HR-ASD group, not the HR-TD or HR-Atyp group. This is consistent with the previous paper that did not find any associations reaching significance in HR infants who did not develop ASD at later age. The ADI-R has been designed to measure atypicalities in RRBs, and might therefore not pick up typical variation within smaller ranges in the HR-no ASD groups. Furthermore, RRBs in infants are relatively low-frequency behaviours which will more likely be reported during the ADI-R where parents report on their child’s behaviour over time, rather than being observed during the brief behavioural capture by the ADOS-2.

This study is the first to replicate a neuroimaging predictor of dimensional variation in ASD symptoms in young infants. Findings from structural and functional MRI studies show converging evidence in support of associations between abnormalities in fronto-striatal circuits and circumscribed interests in ASD. Future directions would be to combine EEG with methods with higher spatial resolution (like MRI or the more infant-friendly NIRS) to unravel the brain systems that underlie functional overconnectivity and circumscribed interests. Further, it will be important to trace whether we can identify early cognitive manifestations of circumscribed interests in infants with ASD that may relate to concurrent hyperconnectivity, for example atypical visual exploration during play^79^, or with eye-tracking methods^23^. Lastly, increased sample sizes would allow for specificity and sensitivity calculations needed for clinical application of this infant neural predictor of phenotypic variation.

## Supplementary information

Supplementary Materials

Supplementary Table S1

Supplementary Table S2

Supplementary Table S3

Supplementary Table S4

Supplementary Table S5

Supplementary Table S6

Supplementary Table S7

Supplementary Table S8

Supplementary Table S9

Supplementary Table S10

Supplementary Table S11

Supplementary Table S12

Supplementary Table S13

Supplementary Table S14

Supplementary Table S15

Supplementary Table S16

Supplementary Table S17

Supplementary Figure S1

Supplementary Figure S2

Supplementary Figure S3

Supplementary Figure S4

Supplementary Figure S5

Supplementary Figure S6

Supplementary Figure S7

Supplementary Figure S8
