# Supplementary material for: Functional EEG connectivity in infants associates with later restricted and repetitive behaviours in autism; a replication study
Source: Transl Psychiatry. 2019 Feb 4;9:66. doi: 10.1038/s41398-019-0380-2 (PMC6361892; doi:10.1038/s41398-019-0380-2)
Supplement: Supplementary file 1 — Supplementary Materials [file 41398_2019_380_MOESM1_ESM.docx]

Supplementary Materials

Title of Main Manuscript:

Functional EEG connectivity in infants associates with later restricted and repetitive behaviours in autism; a replication study

Authors:

Rianne Haartsen^1^, MSc., Emily J.H. Jones, ^1^, PhD., Elena Orekhova ^2,3^, PhD., Tony Charman ^4,5^, PhD., Mark H. Johnson ^1,6^, PhD., & The BASIS team^*^

* The BASIS Team consists of (in alphabetical order): Baron-Cohen, S., Bedford, R., Blasi, A., Bolton, P., Chandler, S., Cheung, C., Davies, K., Elsabbagh, M., Fernandes, J., Gammer, I., Garwood, H., Gliga, T., Guiraud, J., Hudry, K., Liew, M., Lloyd-Fox, S., M.H., Maris, H., O’Hara, L., Pasco, G., Pickles, A., Ribeiro, H., Salomone, E., Tucker, L., and Volein, A..

Affiliations:

^1^ Centre for Brain and Cognitive Development, Birkbeck College, University of London, WC1E 7HX, United Kingdom

^2^ Autism Research Laboratory, Moscow State University of Psychology and Education, Moscow, Russia

^3^ Gillberg Neuropsychiatry Centre, University of Gothenburg, Gothenburg, Sweden

^4^ Department of Psychology, Institute of Psychiatry, Psychology & Neuroscience, King’s College London, De Crespigny Park, London SE5 8AF, United Kingdom

^5^ South London and Maudsley NHS Foundation Trust, Bethlem Royal Hospital, Monks Orchard Road, Beckenham, Kent, BR3 3BX, United Kingdom

^6^ Department of Psychology, University of Cambridge, Cambridge, United Kingdom

*S 1 Methods*

*S 1.1 Participants*

Participants (n = 143) in the current study were recruited as part of the British Autism Study for Infant Siblings (BASIS, [www.basisnetwork.org](http://www.basisnetwork.org)). This study involves multiple visits to the lab during the first years of life (at 4-6, 8-9, 14-15, 24, and 36 months of age). During each visit, infants are assessed with a test-battery of cognitive, neurophysiological, and behavioural measures. Data collection is performed by experienced research assistants. In addition, caregivers/parents fill out questionnaires and are interviewed about the development of their child. Families enrol in the study before the infant is 5 months old. Each infant has an older sibling that is older than 3 years and has an ASD diagnosis (HR younger sibling) or is typically developing (LR younger sibling). Out of the 143 infants in the current cohort, 27 were in the LR group, and 116 were in the HR group. The final sample of children with sufficient clean EEG data consisted of 81 HR infants and 20 LR infants. Research assistants were aware of the familial risk (high or low) of each of the infants while collecting their data at the visits.

Each of the 81 HR infants had at least one older sibling (proband) who has a community clinical diagnosis of ASD (or older half-sibling). Parents of each infant completed the Development and Wellbeing Assessment (DAWBA (1)) and Social Communication Questionnaire (SCQ (2), see S 1.1.1.2). Experienced researchers reviewed results of these questionnaires in order to confirm the diagnoses of the probands. For 66 probands, criteria were met on both the DAWBA and SCQ (total score ≥ 15). For 3 probands, scoring on the SCQ was below threshold, although younger siblings were not excluded due to the proband meeting criteria on the DAWBA and according to expert opinion. Additionally, one proband meeting ASD criteria on the DAWBA did not have a completed SCQ.

Each of the 20 LR infants had at least one older sibling who is typically developing (or half-sibling) and no family history of ASD. These infants were recruited via the volunteer database at Birkbeck Centre for Brain and Cognitive Development. Screening for possible ASD in the older siblings of the LR infants was undertaken using the SCQ, with no child scoring at or above the instrument ASD score of 15. These methods have also been described in a previous study using the same dataset (3).

Characteristics of the participants of the previous cohort are described in the main text and supplementary materials of our previously published report (4).

*S 1.1.1 Rationale for sample sizes*

The samples sizes for the current cohort of the BASIS study were based on findings from the previous BASIS cohort and data pooling, power analyses on existing data, and ASD recurrence rates. Sample sizes for the current cohort were defined prior to the analyses of EEG connectivity on the previous cohort in (4). The previous cohort consisted of 54 HR infants, and 50 LR infants. Increasing samples sizes with respect to the previous cohort and pooling data from both cohorts, increases statistical power to detect effects reaching significance.

The sample sizes for the current cohort were furthermore defined by considering power analyses from the different measures included in the BASIS protocol (fNIRS, eye-tracking, EEG, behavioural assessments). Power analyses from ERP data in 6-month-old infants indicated that 12 infants are sufficient for moderately sized effects, and 25 infants for small sized effects. It was reasoned that for 16 usable infant datasets, data from 20-24 infants would be required to be collected. For fNIRS protocols, similar sample sizes would be needed, while for eye-tracking smaller sample sizes are generally sufficient.

Finally, the recurrence rate of ASD in HR infant siblings is about 20%, while about 10-20% of the infant sibling display atypical outcomes, including broader autism phenotypes. Thus, at least 100 HR infant siblings, and 20 LR siblings would be required for sufficient sizes. Based on these data pooling, power analyses, and recurrence rate, the chosen target sample size for HR infant siblings was 100, and 40 for LR infants. The final sample size for the current cohort was 116 HR infants, and 27 LR infants, which exceed the initial targets.

*S 1.1.1 Clinical assessment*

*S 1.1.1.1 Vineland Adaptive Behavior Scale-II (VABS)*

The Vineland Adaptive Behavior Scale-II (5) is a questionnaire that was given to parent/caregiver in this study. The VABS questionnaire measures adaptive behaviour during daily life of the target individual. Items are scored for 5 domains: 1) Communication, 2) Daily Living Skills, 3) Socialization, 4) Motor Skills, and 5) Maladaptive Behavior (optional). The scores on the different domains yield a composite score for adaptive behaviour. During the visit at 14 months, this questionnaire was given to the parent/caregiver. During the visit at 36 months, this information was collected in the form of a parental interview.

*S 1.1.1.2 Social Communication Questionnaire (SQC)*

The Social Communication Questionnaire (2) measures ASD symptoms in the target child and consists of 40 items. The items are based on the questions from the ADI-R (6). The scores are summed and give an indication on the severity of ASD symptoms.

*S 1.1.2 Outcome diagnosis*

Experienced researchers (TC, GP) reviewed the results of the ADOS-2, ADI-R, VABS, SCQ, and MSEL to determine the outcome diagnosis of each HR infant. Participants who met ASD criteria were assigned to the HR-ASD group (n = 13). The HR-Atyp group consisted of toddlers who were considered atypically developing (n = 21). These toddlers scored either a) above the ADI-R cut-off for ASD and/or above ADOS-2 cut-off for ASD (n = 11), of b) below the value of 77.5 on the MSEL Composite (this value is 1.5 SD below the population mean) or on the MSEL Expressive Language or Receptive Language subscales (n = 6), or c) qualified for both point a and b (n = 4).

Out of 116 HR infants in the cohort, 3 infants had missing outcome data, 64 infants showed typical development, 32 infants showed atypical development, and 17 infants met criteria for ASD. Thus 17 / 113 = 15% of the HR infants in the full cohort met criteria for ASD. In the previous cohort, 32% of the HR infants in the sample met criteria for ASD (17 infants meeting criteria out of 53 HR infants (4)). The recurrence rate for the current sample is lower than the rate for the previous sample. Of note, clinical assessments were supervised by the same team across both cohorts (TC and GP). The recurrence rate in sibling studies is on average 20% in pooled samples (7). There is however substantial variance across samples, largely reflecting sample size. We note that the recurrence rate for the collapsed samples is 20.5% (34 / 166), similar to the recurrence rate of 19% reported by the BSRC (7).

*S 1.1.2.1 Intervention services between 14 and 36 months of age*

Within the UK context, specific interventions for familial at-risk siblings in the first and second year of life are sparse or non-existent. This needs to be understood in the context that the majority of the at-risk siblings were typically developing and few had a community clinical diagnosis of a developmental disorder. We did capture data on access to education and intervention services and very few children received ASD-specific intervention (2 HR-TD infants, 1 HR-Atyp infant, and 5 HR-ASD infants). Further, 13 HR-TD infants, 6 HR-Atyp infants, and 4 HR-ASD infants received more general interventions between 14 months and 36 months, such as community-based activities, services for developmental delay speech and language therapy, occupational therapy, physical therapy, behavioural intervention, special education, or medical treatment. We did not systematically investigate the potential impact of these intervention services on later outcome, since this is a complex and difficult process even in large RCTs, and any attempt to link intervention to outcome in this study would be unreliable, imprecise and underpowered.

*S 1.1.3 Comparisons for age and cognitive level between our previous and current cohorts*

We performed additional analyses to check for differences in age and MSEL scores for the LR, HR-no ASD, and HR-ASD groups between the our prior and current cohort. If the assumptions for normality and homogeneity were both met, parametric independent samples t-tests for means was applied. In the other cases, a non-parametric Mann-Whitney U-test was applied for comparisons between the measures for the prior and current cohort. The results are displayed in Supplementary Table S1.

In the LR group, infants in the prior cohort were assessed at younger age at both the 14 and 36 month visit (*p’*s ≤ 0.049), whereas there were no significant differences were observed between cohorts for cognitive levels measured by MSEL scores (*p’*s ≥ 0.218).

In the HR-no ASD group, infants in the prior cohort displayed younger ages than the current cohort at both visits, (*p’*s ≤ 0.005), whereas cognitive levels measured by MSEL scores were similar between cohorts (*p’*s ≥ 0.287).

In the HR-ASD group, no significant differences were observed between cohorts for age of assessment or cognitive levels measured by the MSEL (*p’*s ≥ 0.214).

*S 1.1.4 The combined HR sample and subtypes of restrictive and repetitive behaviours*

In explanatory analyses we aimed to investigate underlying mechanisms of the association between functional connectivity across selected connections and RRBs. To increase our statistical power, we collapsed the data of HR infants for these analyses from the current cohort with our previous cohort from our previous study (by Orekhova and colleagues (4)). Before combining the datasets, we explored the correlations between functional connectivity and subtypes of RRBs in the cohorts separately. Phenotypic characteristics of the current cohort can be found in Table 1 in the main text, whereas those for the previous cohort can be found in the supplementary materials of the previous report (4).

Scores for the subtypes of restrictive and repetitive behaviours on the ADI-R were calculated from ever scores on specific items on the interview (for the current cohort see Supplementary Table S2). We chose to use ever scores here as the ADI-R ‘Behaviours/Repetitive Interests Algorithm Total’ in our previous and current study is based on the ever scores as opposed to current scores. Ever scores rate the highest severity of the symptoms in the past, whereas current scores rate the severity of the symptoms during the last 3 months. Scores were computed for Repetitive Motor Behaviours, Insistence on Sameness, and Circumscribed Interests subtypes of RRBs. Repetitive Motor Behaviours compromise of the sum of the raw scores on item 69 (Repetitive use of objects), item 77 (Hand and finger mannerisms), and item 78 (Other complex mannerisms and stereotyped body movements). Insistence on Sameness consists of the sum of the raw scores on item 70 (Compulsions and rituals), item 74 (Difficulties with minor change in personal routine or environment), and item 75 (Resistance to trivial changes in the environment). The Circumscribed Interests subtype involves the raw scores on item 67 (Unusual preoccupations), item 68 (Circumscribed Interests), and item 76 (Unusual attachment to objects). Items are scored with 0, 1, 2, 3 reflecting severity, or with 7, 8, or 9 reflecting that the items was not asked or not applicable. If a child had a score of 7, 8, or 9 for a particular item, the child was excluded for the analyses of that subtype. For example, in case the score on item 77 was 9 for a child, this child was not included in the analyses for the Repetitive Motor Behaviours subtype.

Means, standard deviations, minima and maxima of the scores on subtypes of RRBs in the previous cohort, and current cohort are displayed in Supplementary Tables S3, and S4, respectively. Characteristics for the combined complete HR sample can be found in Supplementary Table S5, and summaries for the scores for total and subtypes of RRBs are displayed in Supplementary Table S6.

Overall, HR-no ASD infant scores on the total and subtypes of RRBs display lower medians and variability than scores of HR-ASD infants. Furthermore, scores of HR-no ASD infants are distributed across a lower range than the scores of the HR-ASD infants.

*S 1.2 EEG recording and preprocessing*

EEG coding and preprocessing were done by the researcher (RH) without knowledge of familial risk or later outcome. Data on familial risk and later outcome data were matched with the EEG data for further statistical analyses after data cleaning and preprocessing steps were completed.

*S 1.2.1 Behavioural coding during EEG recording*

Attention was coded from the first frame the child was looking at the screen to the last frame the child was looking at the screen. Interference was defined as any behaviour that is distracting the infant from looking at the screen that would not show up in the EEG signal, and has a minimal duration of 1 second. Interference was coded from the first to the last frame the behaviour was present. Examples of interference are the following: a parent or the experimenter talking to the infant, pointing to the screen to redirect the infant’s attention, or stroking the infant.

*S* *1.2.2* *EEG preprocessing*

The continuous EEG data were first filtered for visual inspection with a high-pass 1 Hz filter and a 48-52 Hz band stop filter. The segments where the child was not looking at the screen and those where there was interference present were marked at bad in the continuous EEG data. Data were visually inspected and episodes with artefacts from muscles, blinks, movement, or electrodes loosing signal were marked as bad data segments. Furthermore, bad channels on the outer side of the net were discarded (E17, E48, E49, E73, E81, E88, E113, E119, E125, E126, E127, and E128) (see Supplementary Figure S1). Channels that were bad for individual participants were also marked. The raw unfiltered data for these marked channels were interpolated before using the average reference. The data were then filtered with a high-pass 1 Hz filter.

Data segments that had not been marked as bad were cut into 1-second epochs with 50% overlap. Another round of automatic data cleaning followed. The EEG signals for the epoch were interpolated if the signal exceeded a threshold of 150 mkV or showed a jump of more than 100 mkV in 4 ms. Epochs were only interpolated when these events occurred in less than 15% of the channels. If interpolation failed because of an insufficient amount of neighbours, epochs were rejected from further analyses. A second round of visual artefact rejection was done to ensure the 1-second epochs contained no bad data. 1-second epochs that still contained bad data were rejected.

At this point the data were collapsed across conditions. Infants with more than 120 clean epochs across conditions were included for further analyses. For the analyses of functional EEG connectivity in infants, a significant amount of data is required. We decided to use the same cut-off of 120 1-second trials as Orekhova and colleagues (4). Collapsing the data across trials results in a higher amount of data per infant and allows for higher inclusion rates compared to when data are not collapsed across conditions.

Fast Fourier Transform (FFT) with a Hanning window was applied to the clean epochs. The complex Fourier values were obtained for each epoch, for each channel, and for each frequency between 0 and 250 Hz (N epochs x 116 channels x 251 frequencies). These values were averaged across the frequencies of interests and thereafter used for the calculations of spectral power and functional EEG connectivity. The frequency of interest will be determined by the results of spectral power analyses.

*S 1.2.3 Spectral power analyses*

It is highly recommended to do spectral power analyses in addition to connectivity analyses (4,8). The reason for this is that is facilitates the choice of an appropriate frequency band of interest. In addition, it allows separating differences in spectral power from differences in connectivity and networks. To this end, we calculated the spectral power by squaring the absolute values of the FFT values for each epoch before calculating the average over trials. Finally, the values were log transformed using the log function in Matlab and averaged across all channels. The frequency band of interest for alpha was chosen based on visual inspection of the spectral power spectrum for 0 to 30 Hz. We expected to see a peak for the alpha rhythm between 7 and 8 Hz as in the previous study (4).

In additional analyses, we investigated any potential differences in posterior and central alpha, and sensory mu oscillations. The average power values for the alpha band were calculated over posterior electrodes over the left and right hemisphere (E58, E59, E64, E65, E66, E69, E70, E71, and E74 for left, and E76, E82, E83, E84, E89, E90, E91, E95, and E96 for right hemisphere electrodes). Average power values were also calculated for the central mu rhythm over central left and right electrodes (E29, E30, E35, E36, E37, E41, and E42 for left, and E87, E93, E103, E104, E105, E110, and E111 for right hemisphere electrodes). Results for these measures are discussed in S 2.2.

*S 1.2.4 Functional connectivity measures*

The phase lag index (PLI) measures consistent, non-zero phase lags by quantifying the asymmetry of the distribution of the phase differences between 2 signals (9).

$PLI=|E \left\{ sgn\left( \mathfrak{I}\left\{ X \right\} \right) \right\}|$,

where I{X} is the imaginary component of the cross-spectrum, and E{.} is the expected value operator (10). Asymmetry arises when the likelihood of a phase difference between -π and 0 degrees is different from the likelihood of a phase difference between 0 andπ. The PLI ranges from 0 to 1. A value of 0 reflects no coupling or coupling with a phase difference of 0 or π and low connectivity, whereas a value of 1 reflects perfect coupling with a phase difference that is not 0 or π and very high connectivity.

The PLI is less sensitive to volume conduction than the measure of coherence. The latter has often been used in other studies (11,12). Volume conduction arises from common pick up from one source by multiple electrodes and by the spread of the electrical field across the scalp (13). Connectivity values for electrodes with short distances will be overestimated as a result of the volume conduction. It is difficult to disentangle whether the connectivity arose from true connectivity between sources or from volume conduction artefacts.

The PLI assumes that non-zero phase lags are more likely to reflect true connectivity, as volume conduction effects cannot account for non-zero phase lags from a single source. Results from model simulations show that the PLI is less sensitive to the effects of common sources than phase coherence and the imaginary part of coherence. Furthermore, the PLI is less affected by different montages than the phase coherence (PC) when applied to EEG recordings. Lastly, spatial patterns showed high PC values over short distances and almost 0 values over long connections. This difference between long and short connections was almost absent for PLI values. This suggests that the PLI is less sensitive to signal spread than PC. By focusing on non-zero phase lag, the PLI is less influenced by volume conduction effects than phase coherence (9).

The PLI is less sensitive to volume conduction and therefore gives a better indication of true connectivity compared to coherence measures. However, the PLI is sensitive to noise when the phase difference lies around 0 or 180°. A small amount of noise can turn a phase lead into a lag and vice versa. The weighted PLI (WPLI) weights the phase lag index values to account for this, and is thus less sensitive to noise than the PLI. The WPLI weights the *sgn(*$\mathfrak{I}$*{X})* by the magnitude of the imaginary component *|*$\mathfrak{I}$*{X}|.* The WPLI is calculated as the following:

$$WPLI= \frac{|E\left\{ \mathfrak{I}\left\{ X \right\} \right\}|}{E\{\left| \mathfrak{I}\left\{ X \right\} \right|\}}= \frac{|E\left\{ \left| \mathfrak{I}\left\{ X \right\} \right|sgn\left( \mathfrak{I\{}X \right) \right\}|}{E\{\left| \mathfrak{I}\left\{ X \right\} \right|\}},$$

where I{X} is the imaginary component of the cross-spectrum, and E{.} is the expected value operator. Phase lags with differences closer to 0 or π will be assigned a very small weight, whereas differences closer to ½ π or -½ π receive the largest weights. Thus, the WPLI is less affected by small phase lags or leads close to the real axis that are easily turned into leads or lags respectively than the PLI.

Both the PLI and WPLI are sensitive to the amount of epochs over which the values are averaged (10). PLI and WPLI values tend to be overestimated for a small number of epochs. The debiased WPLI is another version of the WPLI that less influenced by this bias to the amount of epochs. The debiased WPLI is calculated as follows:

$$dbWPLI= \frac{\sum_{j=1}^{N} \sum_{k\neq j} \mathfrak{I\{}X_{j}\mathfrak{\}I\{}X_{jk}\}}{\sum_{j=1}^{N} \sum_{k\neq j} \mathfrak{|I}\left\{ X_{j} \right\}\mathfrak{I}\left\{ X_{jk} \right\}|}$$

We chose to use the dbWPLI as a measure for functional EEG connectivity in the current study. First, because this measure has been successfully used in a previous study. Second, due to the debiasing method and weighting method the dbWPLI method is more robust in the context of noisy infant data that are unlikely to include a large amount of clean epochs. The dbWPLI values were calculated from the FFT values for each epoch, and then averaged over all available epochs per individual. The dbWPLI values are organized in a connectivity matrix where each row represents the dbWPLI values between 1 channel and each of the 115 other channels. The connectivity matrix for the dbWPLI is mirror around the diagonal as the dbWPLI measures undirected connectivity.

A connectivity matrix for the frequency band of interest is obtained by averaging the connectivity matrices across frequencies. Additionally, averaging all values under the diagonal then gives a global dbWPLI value. Connectivity values for each channel are calculated by averaging the values across the columns excluding the values on the diagonal, e.g. (dbWPLI^E1 – E2^ + dbWPLI^E1-E3^ + … + dbWPLI^E1-E116^)/115. In the specific analyses for the brain and behaviour correlations, we were interested in connections between specific electrode pairs and therefore used a mask for the connectivity matrix. Connections for electrode pairs that were not included in the mask were set to 0. Global connectivity values for the selected connections for each electrode were calculated by averaging the non-zero values across the columns (also see Figure 1). Global connectivity for the selected regions for each infant was calculated by averaging all non-zero values.

*S 1.2.5 Statistical analyses*

Before testing for group differences in connectivity, we examined whether the HR-ASD group differed from the LR, HR-TD, or HR-Atyp groups on other variables such as age, gender, behaviour during the EEG recording, or spectral power, because these might relate to later functional connectivity comparisons between groups. The general applied procedure was as follows. A Shapiro-Wilk test was performed to test for a normal distribution. A Levene’s test was used to further test the homogeneity of the variance between groups. If the results of both the Shapiro-Wilk test and the Levene’s test were non-significant in each group, the assumptions for normality and equality of variances between groups had been met. In that case, a parametric independent samples t-test for means was used to compare data between the HR-ASD group and the other comparison groups (LR, HR-TD, and HR-Atyp group). Means and standard deviations were reported.

If one of the results for the Shapiro-Wilk test or the Levene’s test however was significant (*p* < 0.05), the assumptions for a parametric test were considered as not met. In these cases, the non-parametric Mann-Whitney U-test was used to test whether there was a significant difference between the HR-ASD and the comparison groups. Medians, interquartile ranges, and ranges were reported instead of means and standard deviations. These tests were performed with the Statistical Package for Social Sciences (IBM SPSS Statistics, version 22). This procedure of analyses was also applied in the previous study (4).

If there were differences in age, gender, behaviour during the EEG recording, or spectral power between groups, these factors or covariates would be taken into account by applying an Analysis of Variance (ANOVA) or Analysis of Covariance (ANCOVA) to the functional connectivity data.

Behavioural analyses were performed in accordance with the procedure described above. Independent variable was the group (LR, HR-ASD; HR-TD, HR-ASD; and HR-Atyp, HR-ASD). Dependent variables were percentage of looking, percentage of interference, amount of trials in the combined dataset, amount of trials for each condition (toy, hands, social), and proportion of social trials included in the combined dataset.

Spectral power analyses followed the same method as analyses as the behavioural analyses with the exception of the dependent variables. Dependent variables here were alpha power average across all electrodes, posterior alpha power, left posterior alpha power, right posterior alpha power, central mu power, left central mu power, and right central mu power.

Lastly, additional analyses were done to investigate whether global functional connectivity is associated with age. A Shapiro-Wilk test and Levene’s test were done to determine whether a parametric Pearson’s correlation or a non-parametric Spearman’s correlation would be appropriate.

Functional connectivity analyses were separated into 3 steps of analyses. First, functional connectivity matrices for HR-ASD and comparison groups were compared using the Mann-Whitney U-test with the Network Based Statistics program (NBS) (14). Network Based Statistics (NBS) uses permutation testing to test for differences in networks embedded in connectivity matrices (14). The advantage of permutation testing is that it avoids the multiple comparisons problem. The multiple comparisons problem arises when values for each connection pair are tested in large networks and the probability of a false positive, or Type I error, increases with the increasing amount of tests that are being done. Permutation testing works in 4 steps: 1) the statistical test of the null-hypothesis is tested for every connection in the connectivity matrix A; 2) a chosen test-statistic threshold defines supra-threshold connections; 3) clusters are identified from the supra-threshold connections are a close in the topological space, these clusters are called components; 4) a FWER (family wise error rate)-corrected p-value is calculated for each component with permutation testing.

Permutation testing assumes that if there is no difference between groups or conditions, the data belonging to each group or condition can be randomly assigned to a different group or condition without changing the test-statistic (15). If there would be a difference between the groups or conditions, the test-statistic would be different when the data are randomly assigned to a different group or condition. With each permutation, the data are randomly assigned to different groups or conditions. Then, steps 1 through 3 from the NBS are repeated and the size of the largest component is saved for each permutation. Repeating this permutation process a thousand times creates a null distribution of sizes for the largest component if the null hypothesis was true.

Finally, the size of the component for the actual data is compared with the null distribution obtained during the permutation testing. The FWER-corrected p-value for the actual data is calculated as the percentage of permutations for which the largest component was the same or greater than the size of the component in the actual data (16).

We used the NBS version that utilizes the Mann-Whitney U test as test-statistic, with a one-tailed test and alpha significance level 0.05. The number of permutations was set to 5000. The Z-score threshold used was 1.96. This test was used to test for networks with increased connectivity strengths for the HR-ASD group compared to the other groups.

The second step of the connectivity analyses involved testing for any significant differences on the level of global connectivity in order to support the NBS results. The same statistical procedures used for the behavioural and spectral power analyses were applied to the global connectivity values. Tests for normality and equality of variances were used to establish whether an independent samples t-test or Mann-Whitney U-test would be appropriate to use. For these comparisons, group was between-subjects factor (LR versus HR-ASD; HR-TD versus HR-ASD; HR-Atyp versus HR-ASD; HR-no ASD versus HR-ASD). Dependent variable was global functional connectivity, also called global dbWPLI.

The third step of the connectivity analyses focused on testing for group differences after taking into account age since we found differences in correlations with age between different groups. To this end we used General Linear Model (full factorial) with group (LR, HR-TD, HR-Atyp, HR-ASD) as between-subjects factor, age of EEG assessment in days as covariate, and global functional connectivity values as dependent variable. We ran additional analyses to test the effect of gender on functional connectivity values. An Analysis of Variance (ANOVA) was used to test for an interaction effect between group and gender. Between subject factors were group (LR, HR-TD, HR-Atyp, HR-ASD), and gender (male, female). Lastly, we ran analyses to take into account differences in MSEL composite standard scores. We used a General Linear Model (full factorial) with group (LR, HR-TD, HR-Atyp, HR-ASD) as between-subjects factor, MSEL scores as covariate, and global functional connectivity values as dependent variable.

Finally, analyses for the relations between functional connectivity and the severity of ASD symptoms were performed. For these analyses, we chose to use Spearman’s rank correlations, rather than Pearson’s correlations. Pearson’s correlation assumes that the 2 variables are 2 continuous variables, whereas Spearman’s correlation can also be used with variables that are ordinal and are part of a scale (17). The severity of ASD symptoms in the ADI-R and ADOS-2 are rated on scales, making Spearman’s correlations more appropriate for these analyses than Pearson’s correlations. Moreover, Spearman’s correlation does not assume normality and is less sensitive to outliers (17,18). Correlations were calculated between global dbWPLI and severity of ASD symptoms: a) for the ADI-R, 1) the sum of the Social and Communication Algorithms Total 36m, and 2) Behaviours/Repetitive Interests (RRB) Algorithm Total 36m, b) for the ADOS-2, 1) Social Affect (SA) Total 36m, and 2) Restrictive and Repetitive Behaviors (RRB) Total 36m. Lastly, corrections for multiple comparisons were made using the False Discovery Rate (FDR) in accordance with the Benjamini & Hochberg method for the comparisons within each of the subgroups.

*S 2 Supplementary Results and Discussion*

*S 2.1 Behaviour during EEG recording*

In the current cohort, looking behaviour showed non-Gaussian distributions in the LR, HR-Atyp and HR-ASD group (*p*’s ≤ 0.028), and variances were equal across groups (*p* = 0.327). Mann Whitney U-tests comparing the comparison groups and the HR-ASD group showed no differences between groups for the percentage of looking behaviour during the EEG recording session (*p*’s ≥ 0.355)(see Supplementary Table S7). Interference showed non-Gaussian distributions for each group (*p*’s ≤ 0.047), and variances were not equal across groups (*p* = 0.046). Mann Whitney U-tests comparing the comparison groups and the HR-ASD group showed no differences between groups for the percentage of interference during the EEG recording session (*p*’s ≥ 0.128).

There were no differences in total amount of epochs when epochs across conditions are combined when comparing between the HR-ASD group and other groups (*p*’s ≥ 0.578) (see Supplementary Table S8).

Similar results were obtained for the amount of epochs from the toy condition only, and the hand condition only (*p*’s ≥ 0.128). As for the social condition, the proportion of social epochs in the combined dataset was significantly less for HR-ASD infants compared to the other groups (LR vs. HR-ASD: *U* = 56, *z* = -2.69, exact 2-tailed *p* = 0.006; HR-TD vs. HR-ASD: *U* = 177, *z* = -2.306, asymptotic 2-tailed *p* = 0.021; HR-Atyp vs. HR-ASD: *U* = 70, *z* = -2.357, exact 2-tailed *p* = 0.018; *Mdn*_LR_ = 38, *IQR*_LR_ = 18; *Mdn*_HR-TD_ = 35, *IQR*_HR-TD_ = 12; *Mdn*_HR-Atyp_ = 36, *IQR*_HR-Atyp_ = 10; and *Mdn*_HR-ASD_ = 30, *IQR*_HR-ASD_ = 29). This implies that the comparison groups were more attentive and showed less interference during the social condition than the HR-ASD group. There were no differences between the HR-ASD and comparisons groups for the proportion of overlapping trials (*p*’s ≥ 0.413). A higher proportion of overlapping trials would suggest that the trials were drawn from long periods where the infants was showing attention, rather than many short periods of fleeting attention without interference. This suggests that there was no difference between comparison and HR-ASD groups in the length of the periods of attention and no interference.

The previous study found no differences between the HR-ASD and LR, or HR-no ASD group for behaviours during the EEG (Supplementary data in (4)). To check whether there were any differences in the number of epochs included in the previous cohort, we compared the amount of epochs across all condition, for the toy, hand, and social condition, the proportion of social epochs across all conditions, and the proportion of overlapping epochs. There were no differences between the HR-ASD and the LR or HR-no ASD group for any of these measures (*p*’s ≥ 0.191) (see Supplementary table S9).

Finally, we tested whether there were differences between the cohorts in the percentage of interference because the different behavioural categories for interference coded here compared to those used for the previous cohort (4). Overall, there were no differences between the current and previous cohort for percentages of interference (*U* = 2449, *z* = -1.044, asymptotic 2-tailed *p* = 0.296; *Mdn_Prev_* = 15, *IQR_Prev_* = 14, and *Mdn_Curr_* = 15, *IQR_Curr_* = 20). Comparisons between cohorts within the LR, HR-no ASD, and HR-ASD groups revealed no significant differences either (*p*’s ≥ .451). This suggests that the differences in interference coding categories between the previous and current cohorts likely have small influences on the results of the study. Analyses for the percentages of looking however suggest that infants in the previous cohort were more attentive to the screen than in the current cohort (*U* = 1848, *z* = -3.301, asymptotic 2-tailed *p* = 0.001; *Mdn_Prev_* = 93, *IQR_Prev_* = 8, and *Mdn_Curr_* = 88, *IQR_Curr_* = 10). Looking percentages were similar between cohorts in the LR (*U* = 188, *z* = -1.595, asymptotic 2-tailed *p* = 0.111), and HR-no ASD group (*U* = 490, *z* = -1.295, asymptotic 2-tailed *p* = 0.195), while there was a trend for the HR-ASD group where percentages were higher in the previous than the current cohort (*U* = 37, *z* = -1.736, exact 2-tailed *p* = 0.088; *Mdn_Prev_* = 96, *IQR_Prev_* = 11, and *Mdn_Curr_* = 86, *IQR_Curr_* = 10). There were however no differences in the proportion of overlapping epochs between cohorts (*U* = 2938, *z* = 0.792, asymptotic 2-tailed *p* = 0.428; *Mdn_Prev_* = 75, *IQR_Prev_* = 11, and *Mdn_Curr_* = 77, *IQR_Curr_* = 11). Nonetheless, the total amount of epochs that were included in further analyses was higher for the current than the previous cohort (*U* = 3653, *z* = 3.478, asymptotic 2-tailed *p* = 0.001; *Mdn_Prev_* = 233, *IQR_Prev_* = 155, and *Mdn_Curr_* = 348, *IQR_Curr_* = 191). This was especially true within the group of LR infants (*p* = 0.027), and HR-no ASD infants (*p* = 0.002), whereas the group of HR-ASD infants showed no differences in the amounts of epochs included in the analyses between cohorts (*p* = 0.955) (see Supplementary table S8 and S9 for descriptives of the values for the cohorts).

*S 2.2 Spectral power*

Visual inspection of the spectral power from 0 to 30 Hz confirmed the peak for 7 to 8 Hz, as was the case in the previous study (4) (Supplementary Figure S2). The alpha band in this dataset that would be used in further spectral power and connectivity analyses was defined as 7 to 8 Hz.

All power datasets showed Gaussian distributions (*p*’s ≥ 0.176), and variances across groups were equal (*p*’s ≥ 0.559). T-tests for independent samples were used to compare HR-ASD group with the comparison groups. There were no differences between the HR-ASD and comparison groups for grand average alpha power (7-8 Hz) (*p*’s ≥ 0.720)(see Supplementary Table S10 and Supplementary Figure S2). No differences were observed between the HR-ASD and comparison groups for posterior alpha, posterior alpha left, posterior alpha right, central mu, central mu left, or central mu right (*p*’s ≥ .254).

*S 2.3 Connectivity analyses and potential confounding factors: age, Mullen Scales of Early Learning, gender, and proportion of social epochs included*

It is possible that factors as age, MSEL scores, gender, and the proportion of social epochs included are influencing our connectivity results. To this end, we performed additional analyses to correct for these factors when they were found to be different between groups. Age showed a Gaussian distribution for the LR group only, not for the whole sample, HR-TD, HR-Atyp, or HR-ASD group (*p*’s ≤ 0.049). Global functional connectivity was not related to age in the whole sample (Spearman’s *rho* = -0.04, *p* = 0.69), the LR group (Pearson’s *rho* = 0.02, *p* = 0.92), or the HR-ASD group (Spearman’s *rho* = -0.47, *p* = 0.109). In the HR-TD group, functional connectivity decreased with increasing age (Spearman’s *rho* = -0.33, *p* = 0.023). A correlation in opposite direction was found in the HR-Atyp group where functional connectivity increased with age (Spearman’s *rho* = 0.52, *p* = 0.015; even after removal of the infant with an age of 578 days at the age of assessment, the correlation remained significant, Spearman’s *rho* = 0.45, *p* = 0.047) (Supplementary Figure S3).

The composite standard Scores for the MSEL at the 14-month-old visit showed a normal distribution for each of the 4 groups (*p*’s ≥ 0.282). Global functional connectivity was not related to MSEL scores in the whole sample (Pearson’s *rho* = -0.01, *p* = 0.910), the LR group (Pearson’s *rho* = 0.03, *p* = 0.90), the HR-TD group (Pearson’s *rho* = -0.05, *p* = 0.719), the HR-Atyp group (Pearson’s *rho* = -0.27, *p* = 0.241), or the HR-ASD group (Pearson’s *rho* = 0.34, *p* = 0.249) (Supplementary Figure S4).

As for the proportion of social epochs included in the epochs across all conditions, associations with global functional connectivity reached significance in the HR-TD group (Pearson’s *rho* = -0.35, *p* = 0.015), and the HR-ASD group (Pearson’s *rho* = -0.58, *p* = 0.039), whereas a trend was observed in the HR-Atyp group (Pearson’s *rho* = -0.395, *p* = 0.077). No significant association between global functional connectivity and proportion of social epochs was observed in the LR group (Pearson’s *rho* = 0.17, *p* = 0.474).

Correlations between connectivity and age, and proportion of social epochs were found to be different between groups, whereas they were similar between groups for MSEL scores at 14 months. We performed 4 separate General Linear Models correcting for age, MSEL scores, proportion social epochs, and gender by using the latter as factor and the three former as covariate. Analyses accounting for age showed that there was no significant effect of group after controlling for age, *F*(3,96) = 0.89, *p* = 0.449. The assumption of homogeneity of regression slopes was however not met as shown in the scatterplot (Supplementary Figure S3), and by the interaction between age and group reaching significance, *F*(3,93) = 3.68, *p* = 0.012. This suggests that the results of this analysis should be interpreted with caution.

The analyses accounting for MSEL scores at 14 months yielded no significant effect of group after controlling for MSEL scores, *F*(3,96) = 0.91, *p* = 0.440. Here, the assumption of homogeneity of regression slopes was met, as the interaction term between MSEL scores at 14 months and group did not reach significance (*F*(3,93) = 1.48, *p* = 0.220).

The analyses accounting for the proportion of social epochs showed similar results. The effect of group did not reach significance after controlling for the proportion of social epochs, *F*(3,96) = 1.35, *p* = 0.262. Assumptions for homogeneity of slopes were met as well: *F*(3,93) = 1.55, *p* = 0.206 for the interaction Group x Proportion social epochs.

Lastly, we tested for the effects of gender on functional connectivity. There was a trend effect for gender, *F*(1, 93) = 2.92, *p* = 0.091, but no main effect of group, *F*(3,93) = 0.81, *p* = 0.490. The interaction between group and gender did not reach significance, *F*(3, 93) = 0.40, *p* = 0.754.

These results show that global functional connectivity is similar across groups even when taking age, cognitive ability levels, proportion of social epochs, and gender into account.

*S 2.4 Global connectivity comparisons between the HR-no ASD and HR-ASD group*

In the main manuscript, we compared the HR-ASD group with the HR-TD and HR-Atyp group, whereas Orekhova and colleagues (4) compared the HR-ASD group with the HR-no ASD group, the latter being a combined sample of the HR-TD and HR-Atyp group. To make sure that differences in the design for the current and previous study have not influenced the results, we also compared the functional connectivity measures between the HR-ASD and HR-no ASD group.

First, results from the analyses with the NBS program yielded no differences between networks in the HR-ASD and HR-no ASD group. Second, analyses for global connectivity across all connections with the Mann-Whitney U-test yielded no significant differences between the HR-ASD and HR-no ASD group: *U* = 400, *z* = -0.540, asymptotic 2-tailed *p* = 0.589; *Mdn*_HR-no ASD_ = 0.01, *IQR*_HR-no ASD_ = 0.02, and *Mdn*_HR-ASD_ = 0.02, *IQR*_HR-ASD_ = 0.02.

*S 2.5 Connectivity analyses for selected connections*

The analyses for the global connectivity across all electrodes were repeated using global connectivity across selected connections calculated from the mean for the selected connections only. The selected connections used here were based on the results from the previous study (4).

The assumptions for normality (*p*’s < 0.001) and equal variances among groups were not met (*p* = 0.003). No differences were found in global connectivity for selected connections between the HR-ASD and comparison groups: LR vs. HR-ASD: *U* = 97, *z* = -1.216, exact 2-tailed *p* = 0.235; HR-TD vs. HR-ASD: *U* = 254, *z* = 0.924, asymptotic 2-tailed *p* = 0.355; HR-Atyp vs. HR-ASD: *U* = 115, *z* = -0.762, exact 2-tailed *p* = 0.462; *Mdn*_LR_ = 0.03, *IQR*_LR_ = 0.04; *Mdn*_HR-TD_ = 0.02, *IQR*_HR-TD_ = 0.03; *Mdn*_HR-Atyp_ = 0.02, *IQR*_HR-Atyp_ = 0.06; and *Mdn*_HR-ASD_ = 0.01, *IQR*_HR-ASD_ = 0.04. Furthermore, no differences were found for the comparison between the HR-ASD and HR-no ASD group: *U* = 369, *z* = -0.939, asymptotic 2-tailed *p* = 0.348; *Mdn*_HR-no ASD_ = 0.02, *IQR*_HR-no ASD_ = 0.03, and *Mdn*_HR-ASD_ = 0.01, *IQR*_HR-ASD_ = 0.04.

*S 2.6 Functional connectivity and dimensional traits in HR-TD and HR-Atyp group*

After investigating the correlations between functional connectivity and symptom severity in the complete HR sample and HR-ASD group separately the question arises whether there were any other significant correlations within the other HR groups. The Spearman’s correlation analyses described above where additionally applied to the HR-TD and HR-Atyp group separately (see Supplementary Table S11). A FDR correction was applied to correct for the multiple comparisons made with these unplanned analyses (17). Only 2 significant correlations were found, both in the HR-Atyp group: the correlation between global connectivity and the ADI-R Social and Communication total (Spearman’s *rho* = 0.48, *p* = 0.027), and between global connectivity among selected connections found in the previous study and the ADI-R Social and Communication total (Spearman’s *rho* = 0.49, *p* = 0.023). However, these correlations did not survive after the FDR correction for the 8 comparisons made within each group.

*S 2.7 Functional connectivity and dimensional traits based on ADOS-G scoring*

In our previous study, we used the ADOS-G to measure ASD traits, whereas we use ADOS-2 in the current study. In order to overcome this difference, we investigated correlations between functional connectivity and algorithm scoring according to the ADOS-G based on our ADOS-2 scores. Functional connectivity measures were global functional connectivity averaged across all connections, and functional connectivity averaged across the selected connections found in the previous study (4). The results of these analyses are depicted in Supplementary Table S12.

None of the investigated correlations between functional connectivity and dimensional traits measured with ADOS-G scoring reached significance (*p*’s ≥ 0.304).

*S 2.8 Functional connectivity and dimensions of the MSEL*

We also tested whether functional connectivity across all connections or across selected connections found in the previous study was related to the T-scores on the sub dimensions of the MSEL: visual reception, fine motor, receptive language, and expressive language. The results are displayed in Supplementary Table S13. None of the correlations investigated reached significance in any of the groups (uncorrected *p*’s ≥ 0.081).

*S 2.9 Functional connectivity and subtypes of restrictive and repetitive behaviours*

Possibly, the correlation between functional connectivity and restricted and repetitive behaviours is driven by one of the 3 subtypes of these behaviours or by the difference between current and ever scores. To this end, we examined associations between functional connectivity and subtypes of repetitive behaviours in the complete HR sample, and separately for HR-ASD and HR-no ASD infants. The functional connectivity measures were based on averages across all connections, and averages across the selected connections found in the previous study (4). Before combining the cohorts, we calculated the correlations for our previous and current cohorts separately. The results of these analyses are reported below.

*S 2.9.1 Functional connectivity and subtypes of restrictive and repetitive behaviours in our previous cohort*

Spearman’s correlations between functional connectivity and subtypes of RRBs for our previous cohort are displayed in Supplementary Table S14. Values that remained significant after the FDR correction for the 6 comparisons made within each group are printed in bold. Data are displayed in Supplementary Figures S5 – S8. Caution note: lines in figures represent correlations for the combined sample, not our previous or current sample.

In the HR-ASD group, both global connectivity and connectivity across selected connections were related to circumscribed interests (*p’*s ≤ 0.015). In the HR sample, global connectivity was related to insistence on sameness (Spearman’s *rho* = 0.42, *p* = 0.029). Furthermore, connectivity across selected connections associated with each of the subtypes of RRBs (*p’*s ≤ 0.022). No correlations surviving FDR corrections were observed in the HR-no ASD group.

*S 2.9.2 Functional connectivity and subtypes of restrictive and repetitive behaviours in our current cohort*

Spearman’s correlations between functional connectivity and subtypes of RRBs for the current cohort are displayed in Supplementary Table S15. Values that remained significant after the FDR correction for the 6 comparisons made within each group are printed in bold. Data are displayed in Supplementary Figures S5 – S8. Caution note: lines in figures represent correlations for the combined sample, not our previous or current sample.

No correlations surviving correction for multiple comparisons were observed within any of the HR-no ASD, HR-ASD, or all HR groups.

*S 2.9.3 Functional connectivity and subtypes of restrictive and repetitive behaviours in a combined sample of both cohorts*

Spearman’s correlations and p-values between functional connectivity and RRB measures for the combined sample are displayed in Supplementary Table S16, and data are presented in Supplementary Figures S5 through S8.

In addition to the correlations reported in the main text where both measures of functional connectivity were associated with circumscribed interests in the HR infants, we found one correlation reaching a trend: functional connectivity across selected connections associated on trend level with circumscribed interests in the HR-ASD group (Spearman’s *rho* = 0.38, *p* = 0.086).

*S.2.10 Exploratory analyses using Bayesian inference*

Bayesian inference quantifies the amount of evidence for the null hypothesis (no differences between groups) and for the alternative hypothesis (difference in connectivity between groups), or whether there is no evidence for either hypothesis. In contrast, traditional statistical tests like parametric t-tests and non-parametric Whitney U-tests provide p-values that are used to reject the null hypothesis and accept the alternative hypothesis in case *p* < .05, or to accept the null hypothesis in all other cases. These tests do not take into account the fact that the plausibility of collecting the data under the null hypothesis might be as low as the plausibility of collecting the data under the alternative hypothesis. Bayesian inference can incorporate prior knowledge, and distinguish between evidence for the null hypothesis, alternative hypothesis, and no evidence for either. In situations where traditional statistical tests provide p-values above 0.05, Bayesian inference can inform whether the data provide no evidence for either hypothesis, or whether there is considerable evidence for the null hypothesis (19–21). To this end, we tested whether the data of the combined sample provide evidence for the null hypothesis (no difference in connectivity between the HR-ASD and comparison groups), or no evidence for either hypothesis.

The analyses were performed with the JASP program, version 0.8.6. Bayesian inference versions of non-parametric tests currently still need to be implemented. As a result, we used Bayesian Independent-sample t-test to compare data for the HR-ASD and comparison groups. Data for global connectivity across all connection, and global connectivity across selected connections based on the findings of the previous study (4) were not normally distributed within groups. We therefore first transformed the data using log10 transformation in SPSS, before analysing the data with JASP. We further used Bayes Factor BF_10_, which reflects the amount of evidence for the alternative hypothesis as opposed to the null hypothesis, and has a clearly defined classification scheme (20). Values of a BF_10_ lower than 1/3 provide stronger evidence in support of the null hypothesis (the probability of the alternative hypothesis explaining the data is less than 1/3 times more likely than the probability of the null hypothesis explaining the data), while values above 3 provide stronger evidence in support of the alternative hypothesis. Values ranging between 1/3 and 3 reflect no evidence for either hypothesis. The setting for the prior was the default setting.

Results for the Bayesian inference analyses are presented in Supplementary Table S17. The data suggest that there is more support for the null hypothesis stating no differences between groups for log global connectivity across all the connections. This holds for each of the comparisons: LR vs. HR-ASD, HR-no ASD vs. HR-ASD, HR-TD vs. HR-ASD, and HR-Atyp vs. HR-ASD infants (BF_10_ ≤ 0.310). In contrast, the data were inconclusive for the same comparisons for log global connectivity across the selected connections (0.454 ≤ BF_10_ ≤ 1.678).

*References for Supplementary Material*

1. Goodman R, Ford T, Richards H, Gatward R, Meltzer H. The Development and Well-Being Assessment: Description and Initial Validation of an Integrated Assessment of Child and Adolescent Psychopathology. J Child Psychol Psychiatry [Internet]. Birkbeck College, University of London; 2000;41(5):645–55. Available from: http://doi.wiley.com/10.1111/j.1469-7610.2000.tb02345.x

2. Rutter M, Bailey A, Lord C. The Social Communication Questionnaire [Internet]. Fourth pri. The Social Communication. Western Psychological Services; 2003. Available from: http://www.childhealthcare.org/ug/SCQ/SCQ_Manual-2.pdf

3. Cheung CH., Bedford R, Johnson M., Charman T, Gliga T. Visual search performance in infants associates with later ASD diagnosis. Dev Cogn Neurosci [Internet]. 2016 Sep; Available from: http://linkinghub.elsevier.com/retrieve/pii/S1878929316301219

4. Orekhova E V, Elsabbagh M, Jones EJH, Dawson G, Charman T, Johnson MH, et al. EEG hyper-connectivity in high-risk infants is associated with later autism. J Neurodev Disord. 2014;6(40):1–11.

5. Sparrow SS, Balla DA, Cicchetti DV. Vineland adaptive behavior scales: Survey forms manual. AGS Publ. 2005;

6. Lord C, Rutter M, Le Couteur A. Autism Diagnostic Interview-Revised: a revised version of a diagnostic interview for caregivers of individuals with possible pervasive developmental disorders. J Autism Dev Disord [Internet]. 1994 Oct;24(5):659–85. Available from: http://www.ncbi.nlm.nih.gov/pubmed/7814313

7. Ozonoff S, Young GS, Carter A, Messinger D, Yirmiya N, Zwaigenbaum L, et al. Recurrence risk for autism spectrum disorders: A baby siblings research consortium study. Pediatrics [Internet]. 2011;128(3):e488–95. Available from: http://pediatrics.aappublications.org/content/128/3/e488.full.pdf+html%5Cnhttp://ovidsp.ovid.com/ovidweb.cgi?T=JS&PAGE=reference&D=emed10&NEWS=N&AN=2011490178

8. van Diessen E, Numan T, van Dellen E, van der Kooi AW, Boersma M, Hofman D, et al. Opportunities and methodological challenges in EEG and MEG resting state functional brain network research. Clin Neurophysiol [Internet]. International Federation of Clinical Neurophysiology; 2015;126(8):1468–81. Available from: http://dx.doi.org/10.1016/j.clinph.2014.11.018

9. Stam CJ, Nolte G, Daffertshofer A. Phase lag index: Assessment of functional connectivity from multi channel EEG and MEG with diminished bias from common sources. Hum Brain Mapp. 2007;28(11):1178–93.

10. Vinck M, Oostenveld R, Van Wingerden M, Battaglia F, Pennartz CM a. An improved index of phase-synchronization for electrophysiological data in the presence of volume-conduction, noise and sample-size bias. Neuroimage [Internet]. Elsevier Inc.; 2011;55(4):1548–65. Available from: http://dx.doi.org/10.1016/j.neuroimage.2011.01.055

11. Righi G, Tierney AL, Tager-Flusberg H, Nelson CA. Functional connectivity in the first year of life in infants at risk for autism spectrum disorder: An EEG study. PLoS One. 2014;9(8):1–8.

12. Keehn B, Vogel-Farley V, Tager-Flusberg H, Nelson CA. Atypical hemispheric specialization for faces in infants at-risk for Autism Spectrum Disorder. Autism Res. 2015;8(2):187–98.

13. Cohen MX. Analizing Neural Time Series Data: Theory and Practise. Cambridge, Massachusetts: MIT Press; 2014. 600 p.

14. Zalesky A, Fornito A, Bullmore ET. Network-based statistic: Identifying differences in brain networks. Neuroimage [Internet]. Elsevier Inc.; 2010;53(4):1197–207. Available from: http://linkinghub.elsevier.com/retrieve/pii/S1053811910008852

15. Maris E, Oostenveld R. Nonparametric statistical testing of EEG- and MEG-data. J Neurosci Methods [Internet]. 2007 Aug 15 [cited 2014 Jul 10];164(1):177–90. Available from: http://www.ncbi.nlm.nih.gov/pubmed/17517438

16. Zalesky A. Reference Manual for NBS Connectome (v1.2). 2012;

17. Field A. Discovering statistics using IBM SPSS Statistics. 4th ed. Carmichael M, editor. London: SAGE Publications Ltd; 2014.

18. Rousselet GA, Pernet CR. Improving standards in brain-behavior correlation analyses. Front Hum Neurosci [Internet]. 2012;6(May):119. Available from: http://www.pubmedcentral.nih.gov/articlerender.fcgi?artid=3342588&tool=pmcentrez&rendertype=abstract

19. Wagenmakers EJ, Marsman M, Jamil T, Ly A, Verhagen J, Love J, et al. Bayesian inference for psychology. Part I: Theoretical advantages and practical ramifications. Psychon Bull Rev. 2017;1–23.

20. Wagenmakers EJ, Love J, Marsman M, Jamil T, Ly A, Verhagen J, et al. Bayesian inference for psychology. Part II: Example applications with JASP. Psychon Bull Rev. 2018;25:58–76.

21. Dienes Z. Using Bayes to get the most out of non-significant results. Front Psychol [Internet]. 2014;5(July):1–17. Available from: http://journal.frontiersin.org/article/10.3389/fpsyg.2014.00781/abstract

*Supplementary Figure S1.* EEG layout

Layout of the net used with electrodes for the posterior areas and central areas marked in blue and orange, respectively. Electrodes that were discarded because of bad signal in every subject are depicted in red.

*Supplementary Figure S2.* Spectral power for all groups

a) Spectral power (natural log transform) averaged across left posterior (solid blue line), right posterior (solid red line), left central (dashed blue line), and right central (dashed red line) electrodes for 0 to 30 Hz for each group. The alpha band (7-8 Hz) is highlighted in cyan. b) Topoplots for spectral power (natural log transform) for the 7-8 Hz alpha band for each group. N_LR_= 20, N_HR-TD_ = 47, N_HR-Atyp_ = 21, and N_HR-ASD_ = 13.

*Supplementary Figure S3.* Global dbWPLI connectivity in the alpha range and age at EEG recording (in days)

Each asterisk represents an infant: orange for LR infants, black for HR-TD infants, cyan for HR-Atyp infants, and purple for HR-ASD infants. Functional connectivity was not related to age in the whole sample, or LR infants or HR-ASD infants. A positive correlation between connectivity and age was found for HR-Atyp infants, whereas a negative correlation was found for HR-TD infants. R and p values in the boxes reflect values for the lines in the scatterplot (for HR-Atyp infants, LR infants, HR-ASD infants, and HR-TD infant from top to bottom boxes).

*Supplementary Figure S4.* Global dbWPLI connectivity in the alpha range and Composite Standard Scores for the Mullen Scales of Early Learning (MSEL)

Each asterisk represents an infant: orange for LR infants, black for HR-TD infants, cyan for HR-Atyp infants, and purple for HR-ASD infants. Correlations between functional connectivity and MSEL composite standard scores did not reach significance in complete sample nor any of the subgroups (LR, HR-TD, HR-Atyp, or HR-ASD infants). R and p values in the boxes reflect values for the lines in the scatterplot (for HR-ASD infants, LR infants, HR-TD infants, and HR-Atyp infant from top to bottom boxes).

*Supplementary Figure S5.* Global dbWPLI connectivity and subtypes of Restricted and Repetitive Behaviours for HR infants

Each circle represents a HR infant from the previous study. Each asterisk represents a HR infant from the current sample. The left panel displays values for Repetitive Motor Behaviours (a), the middle panel shows values for Insistence on Sameness (b), and the right panel shows values for Circumscribed Interest (c). Lines represent the correlation across HR infants for the combined sample, with r and p values in the upper right corner of each panel.

*Supplementary Fi
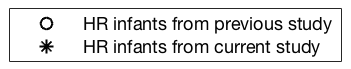
gure S6.* Global dbWPLI connectivity for selected connections and subtypes of Restricted and Repetitive Behaviours for HR infants

Each circle represents a HR infant from our previous study. Each asterisk represents a HR infant from the current sample. The left panel displays values for Repetitive Motor Behaviours (a), the middle panel shows values for Insistence on Sameness (b), and the right panel shows values for Circumscribed Interest (c). Lines represent the correlation across HR infants for the combined sample, with r and p values in the upper right corner of each panel.

*Supplementary Figure S7.* Global dbWPLI connectivity and subtypes of Restricted and Repetitive Behaviours for HR-ASD and HR-no ASD infants

Each circle represents a HR infant from the previous study. Each asterisk represents a HR infant from the current sample. Blue markers reflect HR-no ASD infants, while red markers reflect HR-ASD infants. The left panel displays values for Repetitive Motor Behaviours (a), the middle panel shows values for Insistence on Sameness (b), and the right panel shows values for Circumscribed Interest (d). Blue lines, and left r and p values represent the correlations across HR-no ASD infants for the combined sample. Purple lines, and right r and p values represent the correlations across HR-ASD infants for the combined sample.

*Supplementary Figure S8.* Global dbWPLI connectivity for selected connections and subtypes of Restricted and Repetitive Behaviours for HR-ASD and HR-no ASD infants

Each circle represents a HR infant from the previous study. Each asterisk represents a HR infant from the current sample. Blue markers reflect HR-no ASD infants, while purple markers reflect HR-ASD infants. The left panel displays values for Repetitive Motor Behaviours (a), the middle panel shows values for Insistence on Sameness (b), and the right panel shows values for Circumscribed Interest (c). Blue lines, and left r and p values represent the correlations across HR-no ASD infants for the combined sample. Purple lines, and right r and p values represent the correlations across HR-ASD infants for the combined sample.
