# Supplementary figures and images for: Functional EEG connectivity in infants associates with later restricted and repetitive behaviours in autism; a replication study
Source: Transl Psychiatry. 2019 Feb 4;9:66. doi: 10.1038/s41398-019-0380-2 (PMC6361892; doi:10.1038/s41398-019-0380-2)

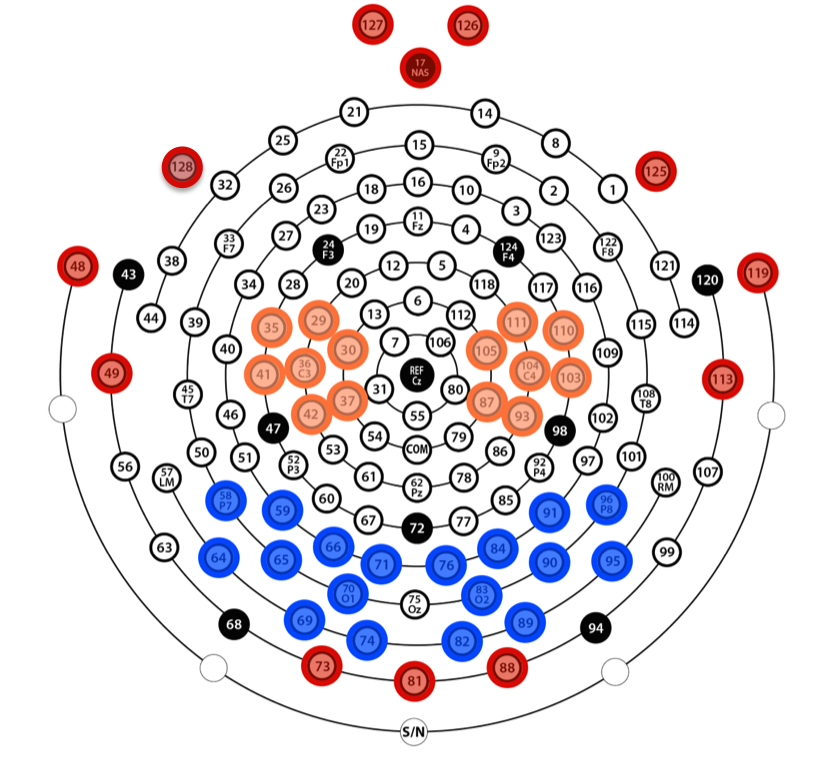

Supplement: Supplementary file 19 — Supplementary Figure S1 [file 41398_2019_380_MOESM19_ESM.tif]

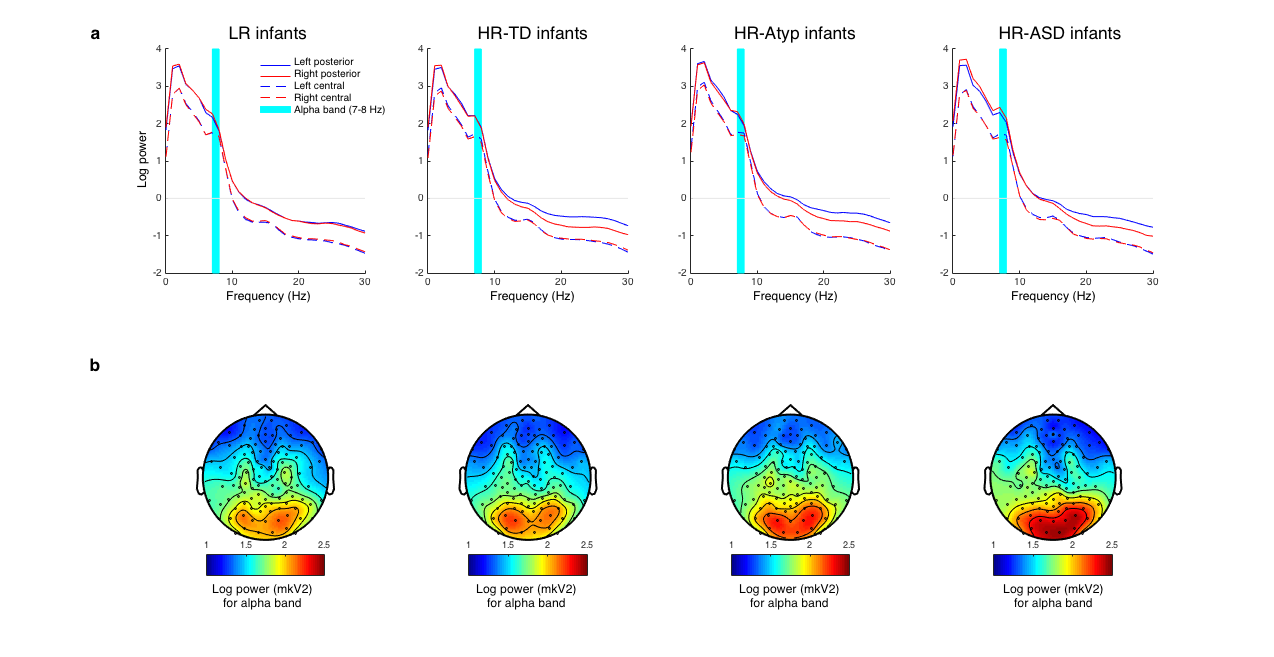

Supplement: Supplementary file 20 — Supplementary Figure S2 [file 41398_2019_380_MOESM20_ESM.tif]

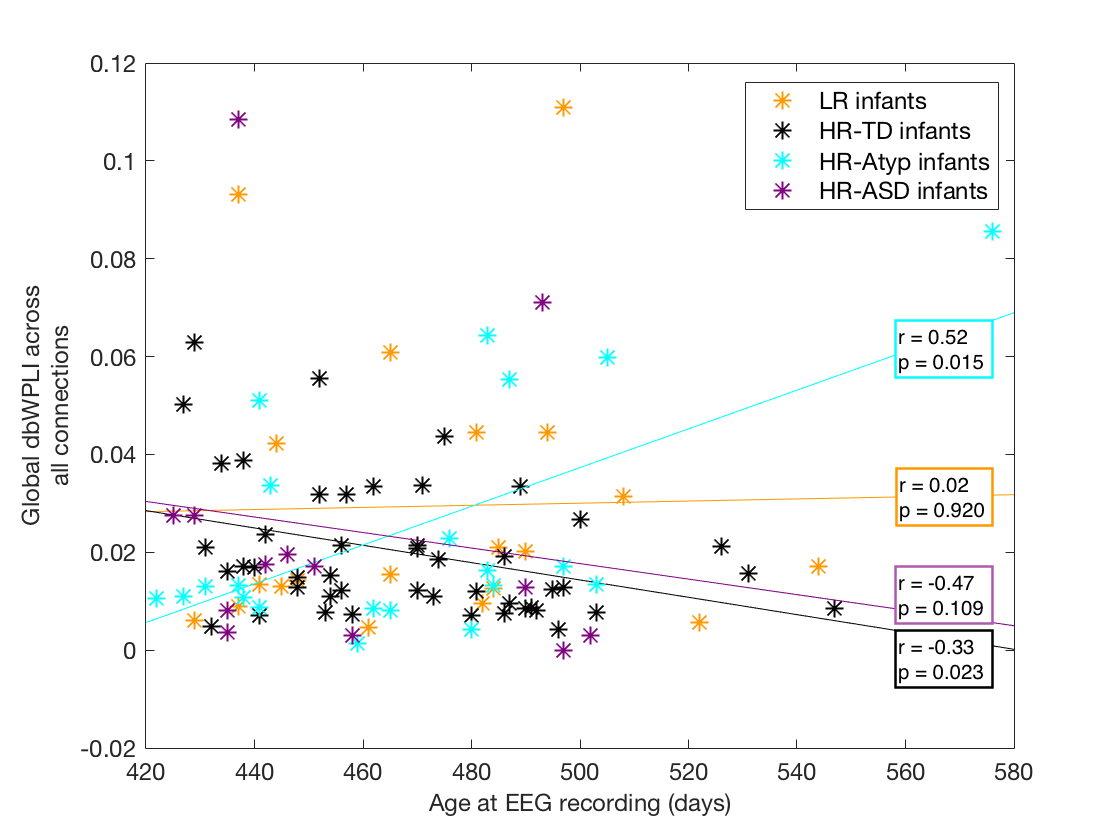

Supplement: Supplementary file 21 — Supplementary Figure S3 [file 41398_2019_380_MOESM21_ESM.tif]

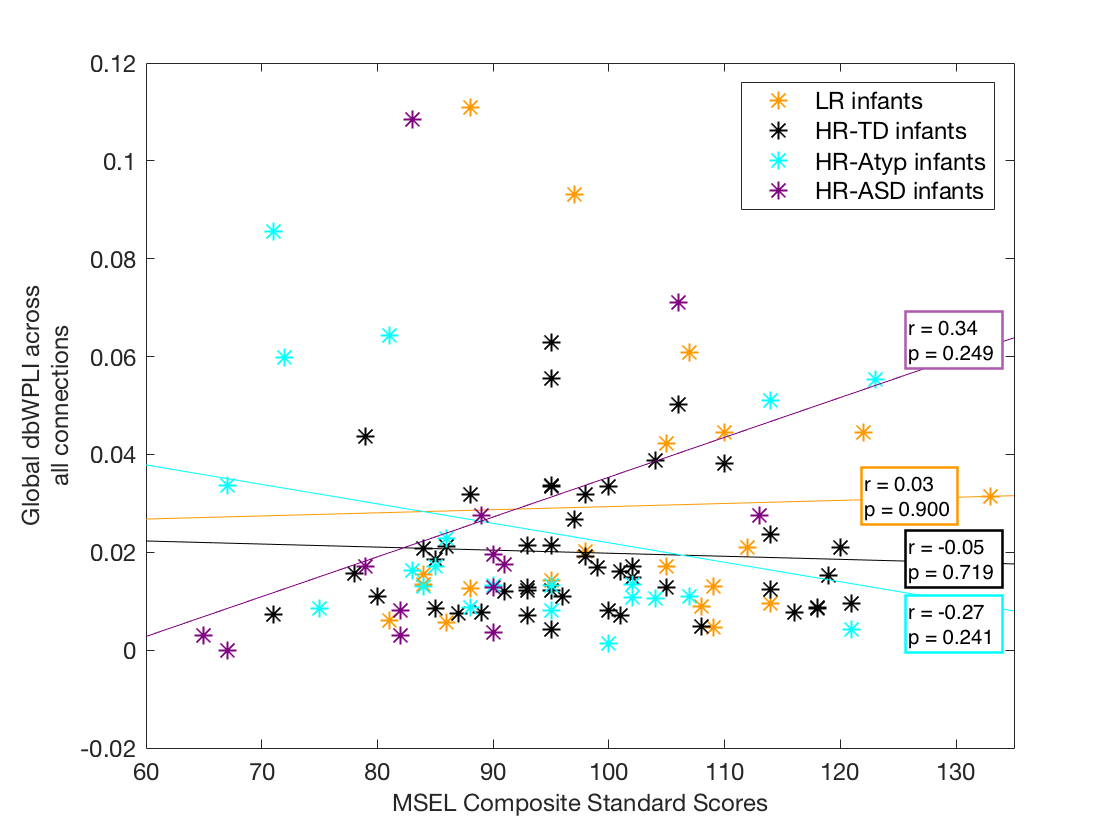

Supplement: Supplementary file 22 — Supplementary Figure S4 [file 41398_2019_380_MOESM22_ESM.tif]

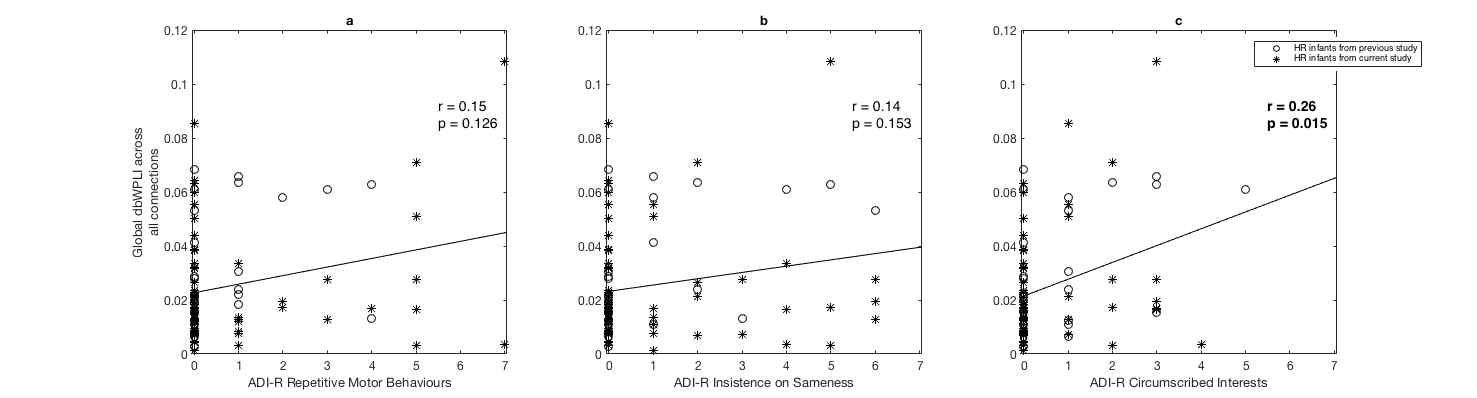

Supplement: Supplementary file 23 — Supplementary Figure S5 [file 41398_2019_380_MOESM23_ESM.tif]

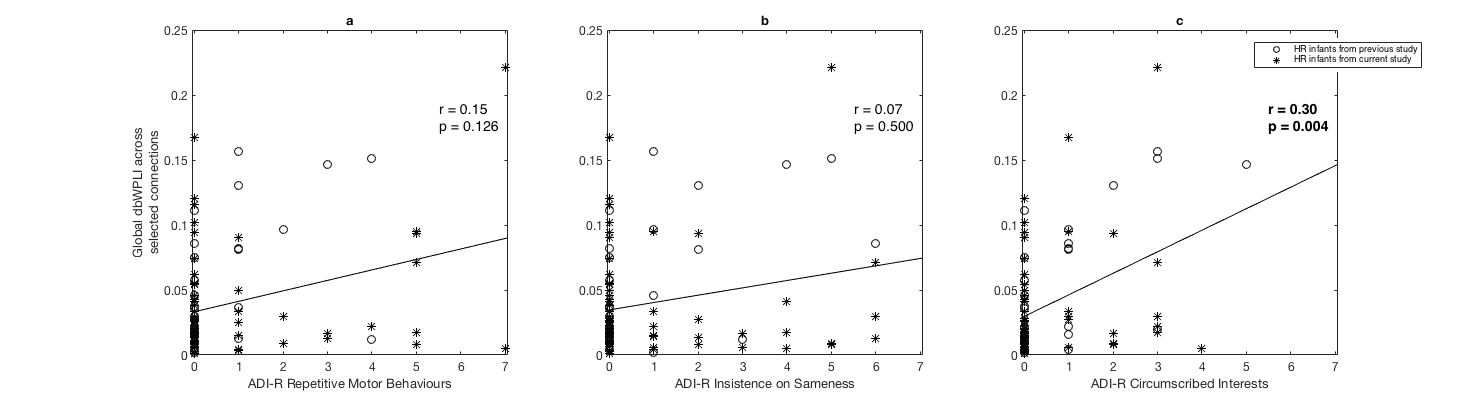

Supplement: Supplementary file 24 — Supplementary Figure S6 [file 41398_2019_380_MOESM24_ESM.tif]

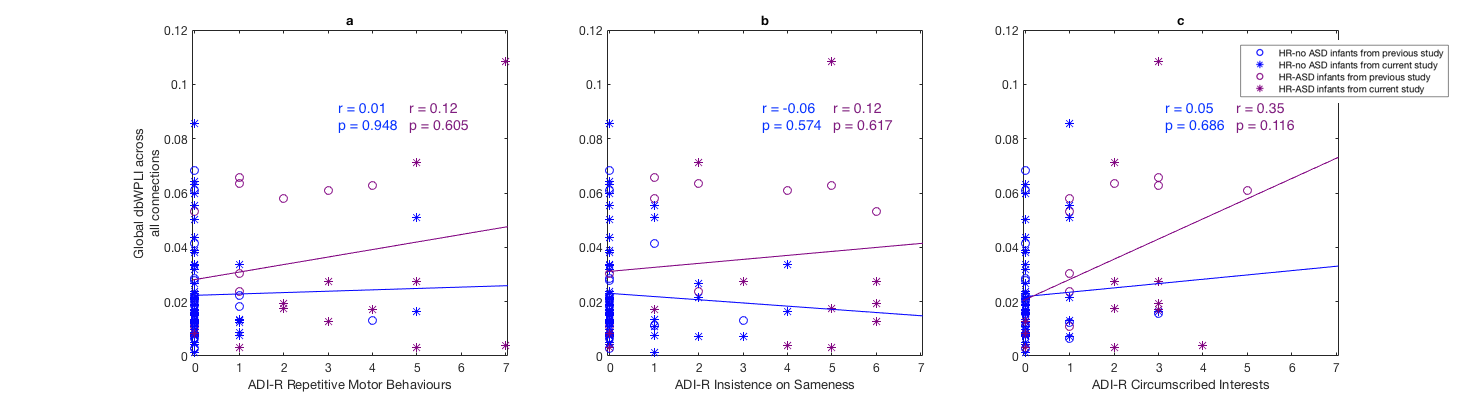

Supplement: Supplementary file 25 — Supplementary Figure S7 [file 41398_2019_380_MOESM25_ESM.tif]

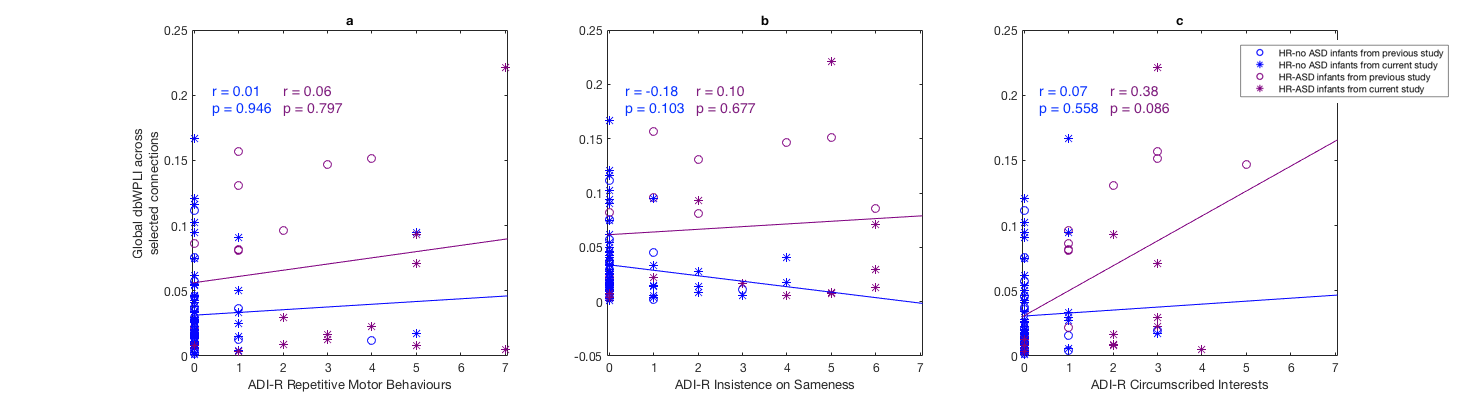

Supplement: Supplementary file 26 — Supplementary Figure S8 [file 41398_2019_380_MOESM26_ESM.tif]
